# Target-Product and Translational Design Principles for Inhalable RNA Nanomedicines

**DOI:** 10.3390/pharmaceutics18080918

**Published:** 2026-07-27

**Authors:** Hossein Omidian, Sumana Dey Chowdhury, Luigi X. Cubeddu

**Affiliations:** Department of Pharmaceutical Sciences, Barry and Judy Silverman College of Pharmacy, Nova Southeastern University, Fort Lauderdale, FL 33328, USA; sd2236@mynsu.nova.edu (S.D.C.); lcubeddu@nova.edu (L.X.C.)

**Keywords:** inhalable RNA nanomedicine, pulmonary delivery, lipid nanoparticles, aerosol formulation, translational pharmacodynamics

## Abstract

Inhalable ribonucleic acid (RNA) nanomedicines are emerging as versatile therapeutics for respiratory diseases and pulmonary metastases, enabling localized delivery of messenger RNA (mRNA), small interfering RNA (siRNA), antisense oligonucleotides, microRNA (miRNA) mimics, self-amplifying RNA, and genome-editing systems. This review synthesizes the available evidence and argues that the field has moved beyond asking whether RNA can reach the lungs. The more consequential translational question is whether RNA cargo, nanocarrier, excipients, manufacturing process, inhalation device, and pulmonary target cell can be integrated into a reproducible therapeutic product. Current research demonstrates progress in disease-corrective mRNA expression, silencing of inflammatory and fibrotic pathways, mucosal vaccination, antiviral therapy, and localized cancer treatment, alongside advances in ionizable lipid nanoparticles, lipid–polymer hybrids, chitosan and polyethyleneimine (PEI) polyplexes, dendrimers, peptide carriers, biomimetic systems, and dry-powder formulations. Translational maturity, however, remains uneven. Many studies demonstrate carrier feasibility, reporter expression, or preclinical activity, whereas fewer establish device-compatible aerosolization, preservation of RNA integrity during processing, traversal of pulmonary barriers, target-cell engagement, repeat-dose tolerability, and clinically meaningful benefit. Development should therefore be target-defined, analytically gated, device-specific, and outcome-centered. Inhalable RNA nanomedicines are best understood as integrated pulmonary products whose success depends on preserving RNA function throughout manufacturing, aerosolization, post-deposition barrier navigation, intracellular delivery, and disease-relevant pharmacodynamic activity.

## 1. Introduction

RNA therapeutics expand drug development through transient protein expression, sequence-specific gene silencing, splice modulation, antigen expression, immune reprogramming, and genome editing [1,2,3,4,5]. For pulmonary medicine, inhalation may increase exposure in diseased airway and alveolar compartments, reduce systemic distribution, and engage epithelial cells, macrophages, fibroblasts, endothelial cells, immune cells, and tumor-associated niches [6,7,8,9,10]. However, the lung is a heterogeneous, immunologically active, mucus- and surfactant-lined interface in which deposition, retention, cellular access, and intracellular RNA bioavailability are distinct events [11,12,13]. The central challenge is therefore not simply lung delivery, but development of a coherent target-product profile linking RNA modality, disease mechanism, pulmonary target cell, formulation architecture, aerosol device, and pharmacodynamic endpoint [11,12,13,14,15].

The strongest rationale occurs when the RNA cargo addresses a defined disease defect or pathogenic pathway. Disease-corrective programs include alpha-1 antitrypsin (A1AT) mRNA for A1AT deficiency, dynein axonemal intermediate chain 1 (DNAI1) mRNA for primary ciliary dyskinesia, granulocyte-macrophage colony-stimulating factor (GM-CSF) mRNA for autoimmune pulmonary alveolar proteinosis [8,16,17], and cystic fibrosis transmembrane conductance regulator (CFTR) mRNA for cystic fibrosis, including the Phase 1/2 trial of aerosolized MRT5005 in adults with severe class I/II CFTR mutations [18,19]. Fibrosis and pulmonary vascular remodeling programs target transforming growth factor beta 1 (TGF-β1), plasminogen activator inhibitor 1 (PAI-1)/C-X-C chemokine receptor type 4 (CXCR4), and microRNA-145 (miR-145) [20,21,22,23]. In inflammation, asthma, and infection, targets include tumor necrosis factor alpha (TNF-α), toll-like receptor 4 (TLR4)/nuclear factor kappa-light-chain-enhancer of activated B cells (NF-κB), GATA-binding protein 3 (GATA3) [24,25,26,27], integrin alpha 5 beta 1 (α_5_β_1_), and severe acute respiratory syndrome coronavirus 2 (SARS-CoV-2) RNA-dependent RNA polymerase (RdRp) [28,29]. Cancer programs target Kirsten rat sarcoma viral oncogene homolog (KRAS), B-cell lymphoma 2 (Bcl-2), myeloid cell leukemia 1 (Mcl-1) [1,4,30,31], polo-like kinase 1 (PLK1), and programmed death-ligand 1 (PD-L1) [32,33]. Pulmonary RNA delivery is therefore increasingly organized around disease-modifying targets and cell-selective pharmacology rather than reporter-gene feasibility alone [4,8,10,17,32].

This expansion has been enabled by diverse nanocarriers, including ionizable lipid nanoparticles (LNPs), cationic lipoplexes, lipid–polymer hybrids [20,34,35], PEI and chitosan polyplexes, dendriplexes, cyclodextrin systems [21,24,36,37], peptide- and surfactant-inspired carriers, extracellular-vesicle-like systems [2,38,39,40], sulfonium lipids, biomimetic membrane-coated particles, and hyaluronic acid (HA)-coated systems [25,41,42]. Rationally defined LNPs provide especially clear formulation evidence because lipid ratios, helper lipid identity, polyethylene glycol (PEG)–lipid content [3,13,15,20], sterol composition, RNA encapsulation, and particle size [18,43,44] are treated as interdependent variables. No carrier class is universally superior: surface shielding or charge density [26,34,45], helper lipid or surfactant interaction [13,46], and polymer modification [47,48] may improve stability or mucus interaction while reducing cellular association, endosomal escape, expression, or tolerability. Pulmonary nanocarrier development therefore requires formulation-specific optimization rather than class-level selection.

The manufacturing process and aerosol device are integral to the therapeutic system. Nebulization, soft-mist aerosolization, pressurized metered-dose inhaler (pMDI) formulation, dry-powder inhaler (DPI) engineering, spray drying, spray freeze-drying, thin-film freeze-drying [15,49,50,51,52], lyophilization, cryomilling, and reconstitution [53,54] can alter nanoparticle size, morphology, aggregation, RNA integrity, encapsulation efficiency, redispersibility, and biological function [51,52,53,54,55]. In several studies, soft-mist, laminar-fluid-ejection, and microfluidic platforms [15,56,57,58,59] preserved mRNA or RNA-loaded LNP integrity more effectively than conventional nebulization, whereas vibrating mesh nebulization produced cargo- and formulation-dependent outcomes [55,60]. Thus, an active nanoparticle suspension is not an inhalable RNA product unless critical quality attributes and pharmacological function survive the intended processing and actuation pathway [12,14,15,55].

After deposition, RNA nanomedicines must navigate human mucus, cystic fibrosis sputum, and mucin-rich media [26,34,61,62], as well as pulmonary surfactant, air–liquid interfaces, and endosomal compartments [13,38,46]. Inflamed alveolar and tumor-associated environments add barriers involving macrophage targeting, oxidative stress, immune suppression, stromal organization, and local retention [2,42,63,64]. HA- and PEG-modified lipoplexes, non-PEGylated lipid–polymer hybrids, charge-reversal nanocomplexes [26,34,63], N-acetylcysteine (NAC)-containing LNPs, surfactant-protein-inspired carriers, and hyaluronic acid–dopamine (HA-DA)-coated systems [38,42,54,65] show that mucus penetration, surfactant compatibility, cellular uptake, and intracellular escape must be assessed together. Regional deposition and durable pulmonary residence [21,43,66,67], reduced systemic escape, and cell-specific target engagement [6,68,69] are likewise separate requirements.

Pharmacodynamic evidence is encouraging but uneven. Inflammatory and acute lung injury models support TLR4, TNF-α, microRNA-155 (miR-155), and related anti-inflammatory pathways [25,42,63,70,71]; fibrotic models support TGF-β1, PAI-1/CXCR4, interleukin-11 (IL-11), and T-box transcription factor 2 (Tbx2) [20,21,23,72,73]; infection and vaccination studies demonstrate antiviral silencing, antibacterial host-factor modulation, mucosal immunization, and encoded-antibody protection [3,5,28,29]; and oncology studies report local tumor suppression, mutation-directed editing [4,33], immune activation, and survival extension [2,32,39,64]. These findings demonstrate disease-relevant pulmonary pharmacology, but clinical translation remains early. MRT5005 established the feasibility of nebulized CFTR mRNA administered in LNPs in humans, yet febrile and hypersensitivity reactions occurred and no beneficial or consistent forced expiratory volume in 1 s (FEV_1_) effect was observed [19]. Reporter expression, lung deposition, and preclinical efficacy therefore indicate scientific progress, whereas translational maturity requires integrated evidence of manufacturability, device compatibility, barrier navigation, target-cell engagement, repeat-dose safety, and clinically meaningful function [11,12,15,19,55].

## 2. Therapeutic and Biological Target-Product Profile

A useful target-product profile for inhalable RNA nanomedicines must define the therapeutic objective before formulation or aerosol performance can be interpreted. Its core elements are the RNA modality, disease mechanism, pulmonary cell compartment that must receive the cargo, and the extent to which the experimental model reflects the intended clinical site of action. Across the available evidence base, the field has progressed from generic lung deposition toward defined biological targets, including epithelial protein replacement, macrophage-directed inflammation control, fibroblast and epithelial remodeling in fibrosis, T-cell modulation in asthma, mucosal vaccination, and reprogramming of tumors or the tumor microenvironment. Translational maturity, however, remains uneven: some programs use disease-relevant targets and advanced models, whereas many remain platform demonstrations based on reporter genes, nonspecific cargoes, or simplified cell systems. Publications, new carrier designs, and preclinical proof-of-concept studies therefore represent scientific progress, but do not alone establish translational readiness.

**Figure 1:**
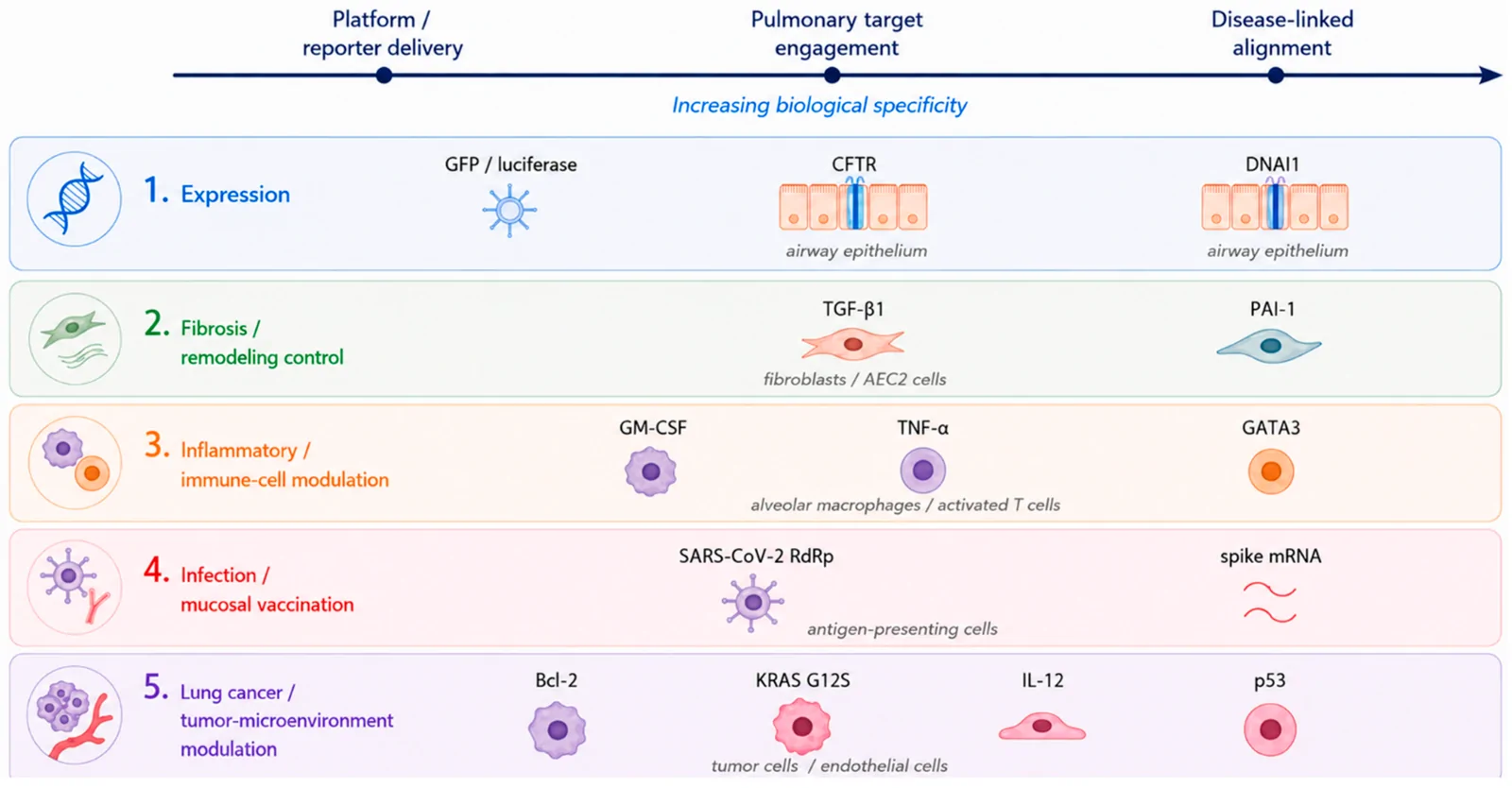
Biological target-product alignment map for inhalable RNA nanomedicines. The schematic organizes biological intent from platform and reporter delivery to pulmonary target engagement and disease-linked alignment. Therapeutic relevance depends on matching the RNA cargo and molecular target to the appropriate pulmonary cell compartment and disease or model context, rather than on lung delivery alone (Prepared with the assistance of Gemini NotebookLM pro and OpenAI ChatGPT Plus).

Figure 1 presents the organizing principle: biological relevance increases when RNA modality, molecular target, intended pulmonary compartment, and disease/model context are aligned. Table 1 summarizes the major target-product profiles across the corpus.

### 2.1. Protein Expression and Replacement by Pulmonary mRNA Delivery

mRNA therapeutics have the clearest biological rationale when the encoded product directly addresses a known disease defect. Protein-replacement or restoration programs include in vitro-transcribed (IVT) A1AT mRNA for A1AT deficiency in human bronchial epithelial cells [16]; DNAI1 mRNA for primary ciliary dyskinesia in primary human bronchial epithelial models and nonhuman-primate lungs [17]; GM-CSF mRNA for aPAP, targeting alveolar macrophages [8]; and CFTR mRNA for cystic fibrosis in inhaled CFTR-deficient animal models and the Phase 1/2 MRT5005 trial in 42 adults with severe class I/II CFTR mutations and baseline percent predicted FEV_1_ (ppFEV_1_) of 50–90% [18,19]. CFTR and DNAI1 mRNA provide the most advanced translational signals because they extend beyond reporter expression to disease-linked cargoes and clinically or physiologically relevant models, although MRT5005 did not produce a beneficial or consistent FEV_1_ effect [17,19].

Other programs use local mRNA expressions to influence repair, fibrosis, inflammation, or antitumor immunity, while reporter studies map access to disease-relevant compartments. Examples include HGF mRNA in emphysema models [43], Tbx2 mRNA in silicosis mice [73], reporter mRNA mapping alveolar epithelial type 2-cell and fibroblast access in bleomycin lung fibrosis [9], IL-11 scFv mRNA in pulmonary fibrosis [72], IL-12 mRNA for lung cancer immunotherapy [39], p53 mRNA for therapeutic tumor modulation [2] and targeted pulmonary expression [10], and DDR1-targeted scFv mRNA combined with PD-L1 siRNA for pulmonary cancer immunotherapy [32]. These studies demonstrate versatility, but robust pulmonary expression does not define a therapeutic product unless cargo, target cell, disease mechanism, and model are coherently aligned. Luciferase- and GFP-encoding mRNA molecules remain useful for carrier development, aerosol compatibility, and compartment mapping, but they are enabling studies rather than disease-specific therapeutic validation [9,10,44,56,68,83].

### 2.2. RNA Silencing and Oligonucleotide Profiles in Fibrosis, Inflammation, and Airway Disease

RNA-silencing and oligonucleotide approaches are strongest when linked to a discrete pathological pathway and specified target compartment. In fibrosis, remodeling, and chronic airway disease, studies converge on TGF-β1, PAI-1, CXCR4/PAI-1 signaling, epithelial sodium channel (ENaC), and NF-κB-associated pathways. TGF-β1 siRNA was studied in bleomycin-induced pulmonary fibrosis, while CXCR4-inhibiting PAI-1 siRNA polyplexes were evaluated in primary lung fibroblasts from fibrotic mice and bleomycin-treated animals [20,21]. Combined CXCR4 inhibition and PAI-1 silencing were assessed in ALI and early- and late-stage IPF [23]. Other profiles include NF-κB siRNA in cystic-fibrosis-relevant mucus, sputum, Calu-3 air–liquid interface cultures, and lipopolysaccharide (LPS)-stimulated 16HBE14o− cells [26]; ENaC-targeting siRNA in triple-cell co-culture and A549 systems [35]; and TGF-β1 siRNA delivery to fibrotic lung lesions [74]. These studies support biologically plausible applications but remain distributed across heterogeneous targets and models rather than a single mature product class.

Inflammatory lung disease is consistently macrophage centered. TNF-α siRNA appears in LPS-induced acute lung inflammation, ALI, pneumonia-oriented inflammation, and inflammatory lung disease studies involving RAW264.7 macrophages, THP-1-derived macrophage-like cells, primary monocytes, alveolar macrophages, and LPS-challenged mice [24,63,71,75,84,85]. Other targets include TLR4 and downstream TNF receptor-associated factor 6 (TRAF6), X-linked inhibitor of apoptosis protein (XIAP), and NF-κB signaling in LPS-induced ALI [25], IL-1β in THP-1-derived macrophage-like cells [77], IL-8 and CXCL-1 in epithelial or rodent inflammation models [76], α_5_β_1_ in Staphylococcus aureus pneumonia [29], and anti-miR-155 in ALI [70]. Repeated use of macrophage and epithelial models strengthens the rationale for local RNA interference (RNAi), although many studies emphasize knockdown feasibility more than target-product maturity.

Asthma, pulmonary vascular disease, and immune-cell targeting add further specificity. GATA3 siRNA was evaluated in activated asthmatic T cells, human precision-cut lung slices, primary human cluster of differentiation 4 (CD4)-positive T cells, and severe-asthma-oriented formulation studies [27,86]. Transferrin receptor-directed siRNA targeted activated T cells, particularly T helper 2 (Th2) cells, in primary human activated T cells and a murine asthma model [87]. Pulmonary arterial hypertension (PAH) was addressed with an ASO against miR-145 in severe Sugen-5416/hypoxia-induced PAH rats treated systemically with three intravenous injections over 5 weeks at 2 mg/kg [22]. RNA delivery to angiopoietin receptor Tie2 (Tie2)-expressing lung endothelial cells was also explored for inflammatory diseases such as chronic asthma and chronic obstructive pulmonary disease (COPD) [7]. These examples shift the objective from anatomical lung delivery toward cell-selective pulmonary pharmacology.

### 2.3. Infectious Disease and Mucosal Vaccine Target Profiles

Infectious-disease applications include pathogen-directed RNAi, host-pathway modulation, and infection-oriented oligonucleotide delivery. For coronavirus disease 2019 (COVID-19), 15 designed siRNA candidates yielded siR-7 against SARS-CoV-2 RdRp, evaluated in viral reporter systems, infected cells, and a Syrian hamster infection model [28]. For bacterial disease, decoy ODN-containing porous particles were studied in *Pseudomonas aeruginosa* lung infection and inflammation through antimicrobial combination testing, cytotoxicity assessment, and in vivo lung-retention studies [66], while α_5_β_1_ siRNA was tested in *Staphylococcus aureus*-induced pneumonia [29]. These approaches respectively target viral replication, host pathways involved in infection progression, and sustained local co-delivery intended to enhance antibacterial therapy.

Mucosal vaccine and antiviral-expression programs extend inhaled RNA beyond treatment of established lung disease. Protective approaches include SARS-CoV-2 spike mRNA polyplexes delivered intranasally to susceptible mice undergoing lethal challenge [5], and mRNA encoding a broadly neutralizing antibody against influenza hemagglutinin in a lethal hemagglutinin 1 neuraminidase 1 (H1N1) challenge model [3]. Enabling platforms include self-amplifying RNA (saRNA) dry powders developed for respiratory vaccination [53], mRNA- and saRNA-loaded LNP mucosal-delivery systems assessed through luciferase expression [58], and nasal LNP-mRNA delivery evaluated using an Alberta Idealized Nasal Inlet deposition model [88]. Formulation stability, mucosal deposition, reporter or antigen expression, and protective immunity represent different evidentiary levels; the target-product profile is strongest when they are connected within one development path.

### 2.4. Lung Cancer, Metastasis, and Tumor Microenvironment Profiles

Oncology applications show broad target diversity and a risk of over-fragmentation. RNAi and antisense strategies targeted Bcl-2, Mcl-1, survivin, KRAS, MRP1/Bcl-2, βIII-tubulin/PLK1, and GAPDH across A549, B16F10, Lewis lung carcinoma, NCI-H358, lung adenocarcinoma, orthotopic lung-tumor, metastatic lung 2,1cancer, and precision-cut lung slice models [1,30,33,62,78,79,89]. Because GAPDH served mainly as a model silencing endpoint in a lung adenocarcinoma cell line and ex vivo precision-cut lung slices, this breadth demonstrates adaptability across oncogenic, resistance, and proof-of-platform pathways, but not convergence around a dominant tumor target, compartment, or translational model [62]. The strongest profiles therefore specify whether the intended effect is direct tumor-cell killing, resistance suppression, mutation-specific editing, or immune-microenvironment modulation.

mRNA, miRNA, and CRISPR-related strategies add more mechanistically ambitious profiles. p53 mRNA was transferred to NSCLC cells through tumor-associated macrophage-derived extracellular-vesicle biology [2], while dual-targeted nanoparticles evaluated p53 and reporter luciferase- or GFP-encoding mRNA molecules in lung-tumor cells and inflammatory macrophages [10]. IL-12 mRNA was studied in orthotopic and metastatic lung tumors involving cancer cells and innate and adaptive immune compartments [39]. DDR1-targeted scFv mRNA was combined with PD-L1 siRNA in orthotopic and metastatic lung cancer models [32]; a miR-34a mimic was evaluated in an orthotopic lung metastasis model involving lung epithelial and tumor cells [64]; and CRISPR-associated protein 9 (Cas9) mRNA with KRAS glycine 12 to serine (G12S)-targeting single-guide RNA (sgRNA) was delivered in A549 cells and an orthotopic luciferase-expressing A549 (A549-luc) lung-tumor mouse model [4]. These studies expand the biological ambition of inhaled RNA oncology, while increasing the need to distinguish tumor access, target modulation, immune remodeling, therapeutic efficacy, and translational progress.

### 2.5. Platform, Reporter, and Pulmonary Compartment Models

A substantial part of the evidence base remains platform oriented. GFP, EGFP, and luciferase reporters were used across A549, H1299-luc, EGFP-expressing H1299, MDA-MB-231-EGFP, transgenic EGFP mice, and other human epithelial or cancer models [47,49,56,61,81,90,91]. NanoLuc luciferase, GAPDH, and model splicing systems supported formulation, compartment-access, or intracellular-function testing in Vero NanoLuc luciferase, lung adenocarcinoma, precision-cut lung slice, and EGFP-654 models [1,62,82]. These models appropriately test delivery competence, expression, and intracellular functionality, but are not equivalent to disease-modifying therapeutics. Studies using cluster of differentiation 44 (CD44)-overexpressing cells, human airway mucus, cystic fibrosis sputum, air–liquid interface cultures, and isolated perfused rat lungs add biological context for pulmonary targeting [13,26,34,80,92]. Precision-cut lung slices and reporter-mouse models provide additional compartment-level evidence without necessarily establishing clinical feasibility [27,47,62,67,91].

This platform literature remains essential because it defines the biological demands that nanocarrier engineering and aerosolization stability must satisfy. Surfactant protein B (SP-B)- and KL4-inspired peptide systems, cyclodextrin nanocomplexes, chitosan systems, dextran nanogels, and oligoethylenimine–hexadecyl (OEI-HD) polyplexes map epithelial access, surfactant compatibility, intracellular delivery, and reporter-gene activity [37,38,46,93,94,95,96]. LNP cargo-comparison studies and dry-powder or nebulized siRNA/mRNA platforms further show how RNA modality, excipient choice, processing, and aerosolization affect pulmonary delivery [13,50,51,52,55,60,97]. Their principal contribution is not clinical efficacy, but clarification of which pulmonary compartments and RNA modalities can plausibly be matched to inhaled nanomedicine designs.

Overall, the field is biologically expansive but translationally uneven. The most convincing target-product profiles combine a disease-linked RNA cargo, a mechanistically appropriate pulmonary target cell, and a model approximating the intended clinical site of action. CFTR, DNAI1, and GM-CSF are relatively well-defined disease-corrective mRNA programs [8,17,18,19], while TNF-α, TGF-β1, PAI-1, GATA3, and SARS-CoV-2 targets are comparatively well-defined RNAi or oligonucleotide programs [20,21,24,27,28]. Selected oncology targets—p53, PD-L1/DDR1, KRAS G12S, and miR-34a—are biologically coherent but heterogeneous [2,4,32,64]. Reporter and platform studies are better suited to establishing delivery feasibility. Nanocarrier engineering and aerosolization stability should therefore be interpreted as enabling requirements whose relevance depends on the biological product profile they support.

## 3. Carrier Architecture, RNA Loading, and Physicochemical Design

**Figure 2:**
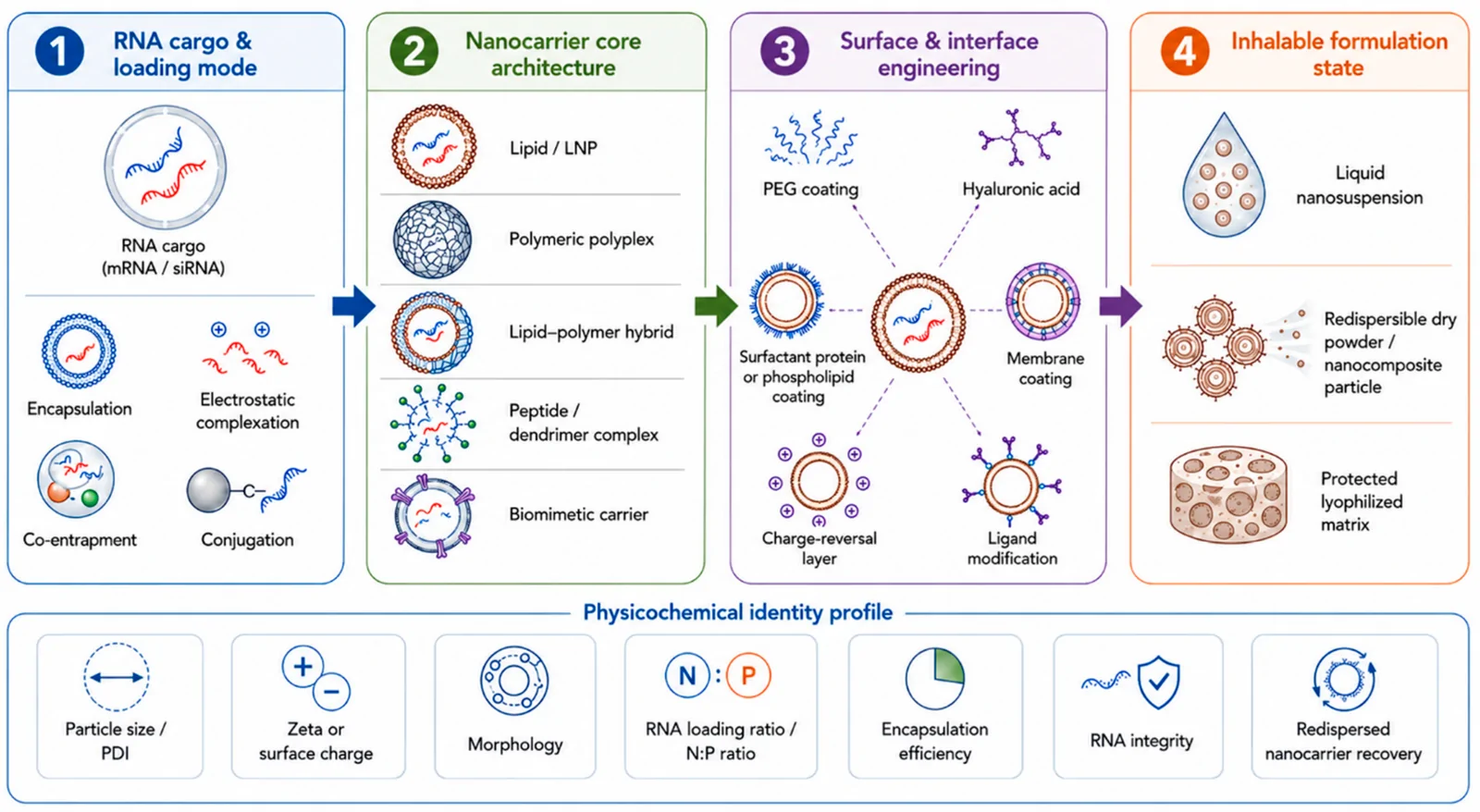
Formulation architecture of inhalable RNA nanomedicines for pulmonary delivery. The figure presents the layered formulation logic from RNA loading to core architecture, surface/interface engineering, and final inhalable state. Loading modes include encapsulation, electrostatic complexation, co-entrapment, and conjugation. Core architectures include lipid/LNP, polymeric, lipid–polymer hybrid, peptide/dendrimer, and biomimetic carriers; surface modifications include PEG, HA, surfactant or phospholipid coatings, charge-reversal layers, and ligands. Final formats may be liquid nanosuspensions, redispersible dry powders or nanocomposites, or protected lyophilized matrices. The accompanying identity profile comprises particle size, PDI, surface charge, morphology, RNA-loading and N/P ratios, encapsulation efficiency, RNA integrity, and redispersed nanocarrier recovery (Prepared with the assistance of Gemini NotebookLM pro and OpenAI ChatGPT Plus).

Inhalable RNA nanomedicines have progressed from relatively simple lipid and polymer complexes to diverse systems designed to maintain RNA association, preserve nanoscale integrity during inhalable formulation, and control the carrier surface presented to airway fluids. Major platforms include ionizable LNPs, lipid–polymer hybrids, PEI- and chitosan-based polyplexes, dendrimer and peptide complexes, surfactant-inspired carriers, and nanocomposite dry powders. The depth of evidence varies considerably: some platforms are supported by quantitative composition–property relationships, whereas others remain proof-of-concept systems with incomplete reporting of particle size, heterogeneity, morphology, RNA encapsulation, surface charge, or post-processing recovery. Because pulmonary formulation imposes constraints not captured by carrier composition alone, Figure 2 links RNA-loading mode, carrier core, surface/interface engineering, inhalable state, and the physicochemical identity profile needed to define a pulmonary RNA formulation.

### 3.1. Lipid-Based Nanocarriers and Ionizable LNP Design

Lipid-based carriers are among the most adaptable systems for inhalable RNA delivery. Cationic liposomes, lipoplexes, and liposome–protamine–DNA complexes generally use electrostatic RNA association and surface modification to tune charge and colloidal behavior. For example, cationic siRNA lipoplexes prepared by ethanol injection were modified with HA and PEG and optimized using HA contents of 10–15% weight/volume (*w*/*v*), PEG at a 5–15 molar ratio, and a positive/negative charge ratio of 4:1 [34]. Liposomal and lipoplex formulations have also delivered mRNA, siRNA, and ASO cargoes through IVT mRNA lipoplexes, neutral and cationic liposomes, 1,2-dioleoyl-3-trimethylammonium-propane (DOTAP)-based lipoplexes, Lipofectamine-based complexes, and dioleoyl-sn-glycero-3-ethylphosphocholine/cholesterol nanoliposomes [6,16,30,60,77,78,98]. These systems establish a practical basis for RNA complexation, although several reports describe carrier type and cargo more fully than quantitative formulation composition.

Ionizable LNP studies provide stronger evidence for rational formulation control because lipid ratios, helper lipids, PEG-lipid content, and RNA-loading parameters are often defined. A TGF-β1 siRNA-LNP used dilinoleylmethyl-4-dimethylaminobutyrate (DLin-MC3-DMA), 1,2-distearoyl-sn-glycero-3-phosphocholine (DSPC), 1,2-dimyristoyl-rac-glycero-3-methoxypolyethylene glycol-2000 (DMG-PEG2000), and cholesterol at 50:10:3:37 with a nitrogen-to-phosphate (N/P) ratio of 3.25 [20]. Other defined systems included TG4C/1,2-dioleoyl-sn-glycero-3-phosphoethanolamine (DOPE)/cholesterol/DMG-PEG2000 at 50:10:38.5:1.5 and AX4/DSPC/cholesterol/DMG-PEG2000 at 35:16:46.5:2.5 [15,43]. Lead Cas9 mRNA/sgRNA formulations included A6 at a 3:1 weight ratio and A8 at a 1:1 weight ratio, with particle sizes below 120 nanometers, near-neutral zeta potential, and encapsulation efficiencies above 80% [4]. Further advances include PEG-free ionizable liposome–mRNA lipocomplexes, sulfonium LNPs, SM-102-based aerosol candidates, onion-like LNP architectures, and ionizable lipidoids identified through chemical-library or computationally guided approaches [41,99,100,101,102].

These studies show that LNP engineering requires coordinated control of ionizable lipid chemistry, helper lipid, sterol, PEG-lipid, buffer, cargo, and processing conditions. The pulmonary PEG dilemma illustrates these interdependencies: PEG-lipid may improve colloidal stability, shear resistance, or mucus penetration, but its optimal content depends on the accompanying formulation, and excessive shielding may reduce cellular uptake or expression [3,18,44,45]. Helper-lipid effects are likewise cargo-dependent, as DOPE- and DSPC-containing LNPs do not behave uniformly with siRNA and mRNA [13]. β-Sitosterol-induced polyhedral morphology promoted endosomal escape, whereas diacylglyceryltrimethylhomoserine (DGTS) substitution preserved particle size and encapsulation efficiency but yielded administration- and assay-dependent transfection activity [18,103]. Sterol and structural-lipid choices therefore alter particle behavior substantially, but do not yet support universal design rules across RNA modalities.

### 3.2. Lipid–Polymer, PLGA-Based, and Emulsion Hybrid Architectures

Hybrid systems address the difficulty of using one material both to associate RNA efficiently and to withstand conversion into an inhalable form. Lipid–polymer nanoparticles distribute these functions across polymeric RNA-containing cores or matrices and lipid shells, additives, or lipoplex-like assemblies. One well-characterized system used a functionalized α,β-poly(N-2-hydroxyethyl)-D,L-aspartamide copolymer containing 35 mole percent 1,2-bis(3-aminopropylamino)ethane, 0.4 mole percent fluorescent dye, and 4.5 mole percent poly(lactic-co-glycolic acid) (PLGA), surrounded by a 1,2-dipalmitoyl-sn-glycero-3-phosphocholine (DPPC)/1,2-distearoyl-sn-glycero-3-phosphoethanolamine–PEG2000 (DSPE-PEG2000) shell. Fresh particles were approximately 164 nanometers, positively charged, and approximately 99% efficient in siRNA encapsulation; after spray drying, spherical particles were approximately 3 micrometers with 80% encapsulation efficiency [81]. PLGA/DPPC hybrid nanoparticles carrying siRNA, with or without PEI, were approximately 150 nanometers with a low polydispersity index (PDI) and a zeta potential near −25 millivolts [35]. Other designs incorporated DOTAP into PLGA matrices or co-entrapped DOTAP/siRNA lipoplexes in PLGA nanoparticles [67,104], while PLGA-based large porous particles and microparticles were developed for ODN or siRNA encapsulation [66,85].

The principal advantage of lipid–polymer hybrids is the combination of nanoscale RNA protection with a second component that can support redispersion, retention, or dry-powder engineering. Their evidence base also reveals a reporting gap: post-processing nanoparticle recovery, residual aggregation, RNA encapsulation, and morphology after aerosolization or drying are not measured consistently. Perfluorocarbon emulsion polyplexes and lipid–membrane hybrids extend the multicomponent design space through fluorinated polymeric CXCR4-antagonist carriers, cholesterol-modified polymeric stabilizers, or apoptotic T-cell membrane interfaces [23,64,74]. Although mechanistically sophisticated, these systems remain difficult to compare without standardized measurements of size distribution, RNA integrity, encapsulation, surface charge, and morphology before and after processing.

### 3.3. Polymeric, Chitosan, Dendrimer, and Peptide Complexation Systems

Polymeric systems primarily use cationic RNA condensation, with PEI and its derivatives forming a prominent class. Reported systems include Alveofact-coated PEI polyplexes characterized by size, PDI, zeta potential, and transmission electron microscopy (TEM) morphology; fatty-acid-modified low-molecular-weight PEI; PEG-modified PEI; other modified PEI derivatives; transferrin- or melittin-modified PEI; and PEI/Bcl-2 siRNA complexes co-formulated with doxorubicin [21,27,31,47,76,80,87]. Their performance depends on chemical modification, shielding, and formulation environment rather than cationic charge alone. Crosslinked oligoethylenimine polyplexes further demonstrate the importance of buffer and excipient context: PEG grafting or PEI–PEG mixing stabilized particles in isotonic saline, whereas Pluronic P68/F68 or Alveofact stabilized them in isotonic glucose, with the Pluronic formulation producing particles as small as 140 nanometers [96].

Non-PEI polymers and cyclodextrins broaden this class while reinforcing the need for standardized characterization. Inulin-vinyl sulfone-graft-poly(2-methyl-2-oxazoline; 1,2-bis[3-aminopropylamino]ethane) [INU-VS-g-(PMeOx;bAPAE)] formed siRNA polyplexes below 30 nanometers at a polymer/siRNA weight ratio of 5:1; SC12CDClickpropylamine formed approximately 200-nanometer cationic siRNA nanocomplexes; and a fast-degrading polyester produced 150–200-nanometer particles with a +15 to +20 millivolt zeta potential and degradation within 4 h at pH 7.4 [37,61,90]. Other platforms include poly(2-dimethylaminoethyl methacrylate)-poly(oligoethylene glycol methyl ether methacrylate) (PDMAEMA-POEGMA) star polymers [33], hyperbranched poly(β-amino ester)s [68], biodegradable poly(amine-co-ester) (PACE) polyplexes [5], functional polyester nanoparticles modified with DMG-PEG2000 or Pluronic F-127 [69], and four-in-one lipidic hyperbranched poly(β-amino ester) materials containing tertiary and quaternary amines, cholesterol, zwitterionic groups, and hydrophobic alkyl tails [73].

Chitosan systems provide a polysaccharide-centered route to RNA association. Piperazine-substituted chitosans formed sub-300-nanometer siRNA complexes at a polymer/siRNA ratio of 5:1 [36]. Guanidinylated O-carboxymethyl chitosan/N-2-hydroxypropyltrimethyl ammonium chloride chitosan nanoparticles contained approximately 16.8% guanidine groups, measured 150–180 nanometers, and had a zeta potential of approximately −17 millivolts [89]. Crosslinked chitosan nanoparticles were below 150 nanometers, positively charged, spherical, non-aggregated at airway pH, and above 96% efficient in encapsulation, with complete siRNA binding at a 50:1 chitosan-to-siRNA ratio [105]. These findings show that chemical substitution and complexation ratio can tune size, charge, and RNA binding, particularly when linked to quantitative particle properties.

Dendrimers and peptides provide more structurally defined complexation motifs. Poly(amidoamine) (PAMAM) and phosphorus dendrimers, triphenylphosphonium (TPP)-modified PAMAM, and the peptide dendrimer KK-46 have complexed siRNA or chemically modified siRNA, with terminal-group chemistry, TPP density, and N/P ratio among the principal variables [24,28,49,84,106]. SP-B fragments, KL_4_, PEGylated KL_4_, SAR(6)EW, and PEGylated pH-responsive peptides demonstrate how peptide sequence, fragment identity, PEG chain length, and amphiphilic balance govern RNA binding and nanoassembly [38,48,65,71,93,95,107]. Molecularly defined examples include PEG(12)KL_4_/mRNA complexes at a 10:1 weight/weight ratio and PEGylated pH-responsive peptide–mRNA self-assemblies using a 12-monomer PEG chain [48,107]. Across peptide and dendrimer studies, however, complexation and functional performance are reported more consistently than a common panel of PDI, morphology, surface charge, and post-processing recovery.

### 3.4. Biomimetic, Surfactant-Coated, and Hierarchical Carrier Designs

Biomimetic carriers shift attention from the RNA-condensing core to the interfaces encountered in the lung. Examples include neutrophil membrane-coated PLGA nanoparticles, extracellular-vesicle-encapsulated IL-12 mRNA, surfactant-interacting dextran nanogels, clinical pulmonary-surfactant-coated nanogels containing functional SP-B, Curosurf-coated nanogels, and HA-DA-coated poly(β-amino ester) vectors [25,39,42,46,108,109]. This approach is relevant because inhaled particles encounter pulmonary surfactants, mucus, airway lining fluid, epithelial surfaces, and immune cells before reaching intracellular targets; stability in buffer therefore may not predict behavior after biological-interface contact.

Evidence supports lung-relevant surface engineering, although physicochemical characterization remains uneven. Clinical pulmonary-surfactant-coated nanogels containing SP-B retained structural integrity and transfection activity after lyophilization, reconstitution, and nebulization [108], while Curosurf-coated nanogels paired pulmonary-surfactant interfaces with β_2_ agonist delivery adjuvants [109]. HA-DA-coated poly(β-amino ester)/siRNA systems combine charge screening with dopamine-mediated bioadhesion and antioxidative functionality [42]. Receptor for advanced glycation end products (RAGE)-directed dual-sensitive transformable (RDDsT) nanocomplexes comprise a cationic siRNA core, charge-reversal polymer shell, and electrostatically adsorbed RAGE-binding peptide responsive to the acidic inflamed alveolar environment [63]. Mesoporous silica systems use electrostatic layer-by-layer crosslinking to embed siRNA and load chloroquine for enhanced endosomal escape [75]. These increasingly sophisticated interfaces remain most defensibly interpreted as tunable carrier designs whose comparability depends on consistent reporting of size distribution, surface charge, morphology, RNA retention, coating stability, and post-processing behavior.

### 3.5. Dry-Powder, Excipient, and Solid-State Formulation Design

Dry-powder and solid-state approaches connect nanocarrier design with manufacturability, storage, and device compatibility. Two broad strategies predominate: direct formulation of naked RNA powders and incorporation of preassembled nanocarriers into protective excipient matrices. Naked siRNA powders were co-spray-dried with mannitol and L-leucine, with 50% weight/weight leucine enriching the particle surface and producing the best aerodynamic performance [50]. High-loading powders containing more than 6% weight/weight siRNA used human serum albumin (HSA) as a dispersion enhancer; increasing HSA increased surface corrugation and preserved up to 78% intact siRNA [97]. Spray-dried EGFP-siRNA/mannitol powders further show that processing variables can change particle morphology, mannitol polymorphism, RNA chemical stability, and transfection capacity [110].

For nanocarrier-containing powders, the key question is whether the original nanocarrier can be recovered with preserved RNA encapsulation and acceptable heterogeneity, not merely whether a respirable particle can be produced. mRNA-LNP powders have used spray freeze-drying with cryoprotecting sugars, hydroalcoholic spray drying with mannitol and leucine, and pH-modified spray freeze-drying to strengthen mRNA–ionizable-lipid interactions while avoiding hygroscopic, deliquescent excipients such as sucrose [51,83,111]. Related saRNA-LNP work used sucrose-containing formulations, freeze-drying, and leucine-assisted cryomilling for respiratory vaccine powder development [53]. Ternary trehalose/dextran/leucine matrices for siRNA-loaded lipidoid–polymer hybrid nanoparticles provide a defined formulation logic: leucine promotes shell formation and dispersion, trehalose protects the nanoparticle surface, and dextran creates porosity and limits trehalose recrystallization [112,113]. A spray-dried LNP-siRNA powder containing a sulfur-containing DLin-MC3-DMA analog, helper lipid, cholesterol, and PEG-DMG in 5% *w*/*v* lactose redispersed to approximately 150 nanometers, maintained residual moisture below 5%, limited RNA loss to below 15%, and retained bioactivity [62]. These measurements link manufacturing, redispersion, and carrier integrity more directly than aerosol output alone.

Accordingly, inhalable RNA formulations should not be judged only by pre-processing particle size or in vitro activity. Characterization should span manufacturing, aerosolization, and redispersion and include particle-size distribution, PDI or heterogeneity, aggregation, morphology, zeta potential or surface properties, RNA integrity, encapsulation efficiency, material recovery, powder moisture, and stability under relevant processing and storage conditions. Studies reporting only carrier identity or biological activity demonstrate scientific progress, but not necessarily translational maturity [11,12]. The most informative platforms connect composition to measurable physicochemical attributes before and after inhalable processing. Table 2 summarizes these design patterns.

Overall, carrier invention and formulation optimization have advanced substantially, but most platforms remain preclinical or formulation-stage technologies, only a small subset has entered clinical testing, and approved inhaled RNA nanomedicines are not established within the summarized evidence. The evidence therefore does not identify a universally superior carrier class. Translationally credible candidates instead combine defined composition, efficient RNA association, reproducible nanoscale properties, stability after aerosolization or drying, and analytical methods capable of detecting processing-induced changes. Device compatibility, aerosol performance, immunotoxicity, in vivo efficacy, and clinical benchmarking should be interpreted only after carrier architecture and physicochemical integrity have been rigorously established.

## 4. Inhalable Dosage Form, Processing Stability, and Device Performance

### 4.1. Dosage-Form Landscape and Product-Enabling Requirements

The central formulation challenge for inhalable RNA nanomedicines is whether a nanocarrier can be converted into a reproducible inhaled product without losing RNA integrity, carrier structure, encapsulation, dispersibility, or biological function. Across the reviewed studies, RNA systems were formulated as nebulized liquids, soft-mist aerosols, microsprayer-administered suspensions, and pMDI or DPI products [36,37,49,56,94]. Solid-state formats included spray-dried, spray-freeze-dried, and TFFD powders; lyophilized and reconstituted formulations; and freeze-dried powders subsequently processed by cryomilling [50,51,52,53,54,62,97]. The most translationally informative studies characterized formulations before and after processing, assessing particle size, polydispersity, aggregation, morphology, RNA integrity, encapsulation efficiency, surface properties, redispersibility, aerosol performance, and retained bioactivity [14,37,50,56,59,62,97].

**Figure 3:**
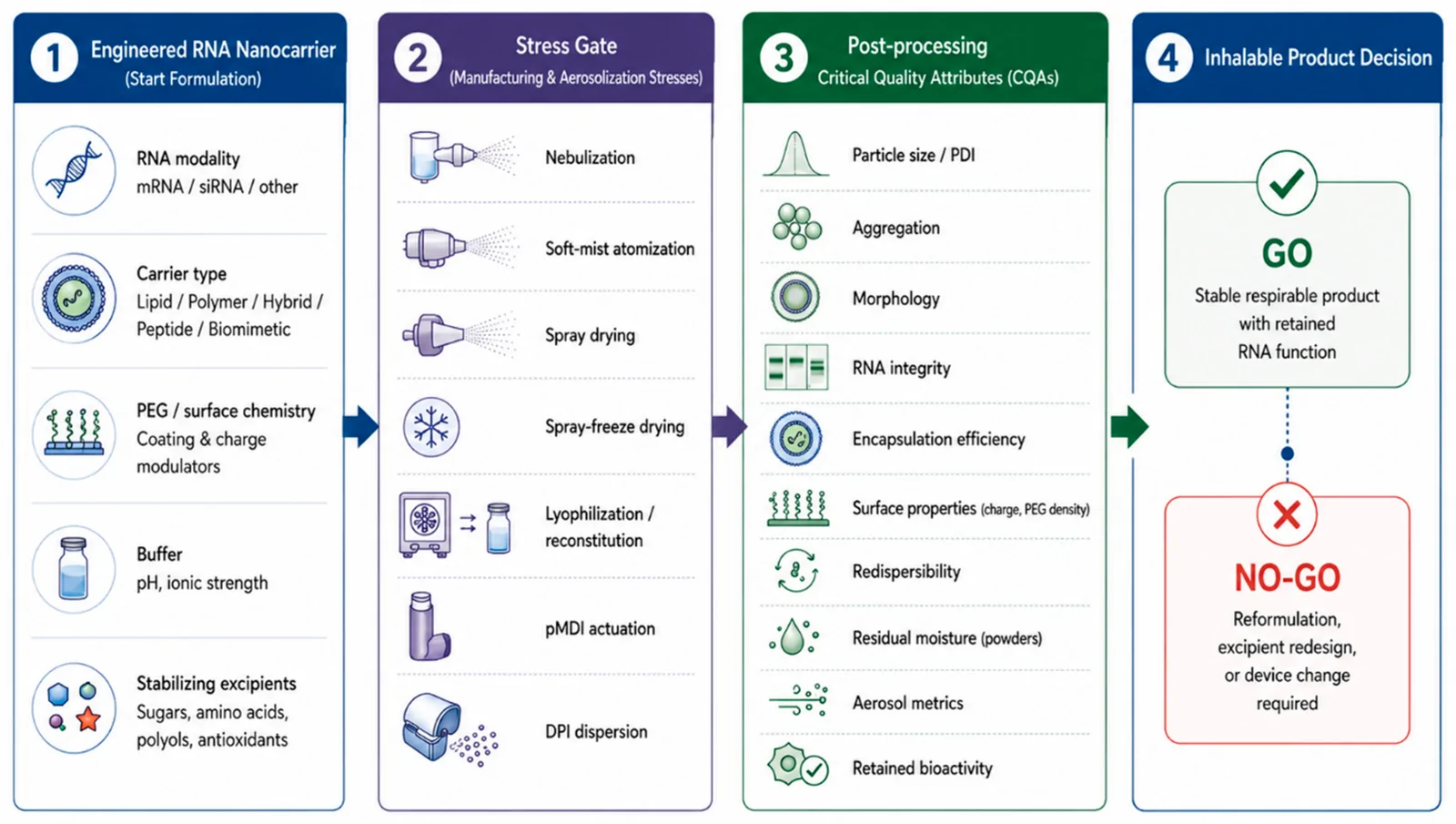
Formulation–process–device coupling in inhalable RNA nanomedicine development. Pulmonary RNA nanocarriers must retain critical quality attributes after manufacturing, storage, aerosolization, and device actuation. Nebulization, spray drying, lyophilization and reconstitution, and device actuation can alter particle size, aggregation, morphology, RNA integrity, encapsulation efficiency, aerosol performance, and biological activity. Product readiness therefore requires experimentally verified compatibility among RNA modality, carrier, surface chemistry, buffer, stabilizing excipients, processing method, and delivery device, resulting in either a stable respirable product or a need for reformulation (Prepared with the assistance of Gemini NotebookLM pro and OpenAI ChatGPT Plus).

The evidence supports a systems-level conclusion: inhalable RNA performance depends on the integrated combination of RNA cargo, nanocarrier, excipient matrix, manufacturing process, and aerosol device rather than carrier chemistry alone. LNPs, polymeric polyplexes, chitosan systems, dendriplexes, peptide complexes, nanogels, lipid–polymer hybrids, and solid lipid nanoparticles have shown pulmonary-delivery potential, but differ substantially in processing tolerance [11,12]. Several siRNA systems retained biological function after nebulization or drying when protected by suitable carriers or excipients, whereas mRNA-LNP formulations often required stabilization against shear, aggregation, encapsulation loss, or cargo degradation [14,37,54,59,104,114,115]. This does not indicate universal superiority of one RNA class; aerosol compatibility must be established for each cargo–carrier–device combination. Figure 3 depicts this formulation–process–device checkpoint.

### 4.2. Liquid Aerosolization and Nebulized RNA Formulations

Nebulized liquids provide a direct route to pulmonary RNA administration but expose nanocarriers to shear, interfacial, and droplet-formation stresses that can destabilize nanoparticle formulations [14]. The most informative studies evaluated aerosolization stability rather than relying only on post-delivery biological outcomes. The LOOP inhaled LNP platform used an excipient-assisted nebulization buffer to protect against shear-associated damage [72]. Design-of-experiments optimization produced stably aerosolized siRNA-LNPs with a DLin-MC3-DMA/DSPC/DMG-PEG_2000_/cholesterol ratio of 50:10:3:37 and an N/P ratio of 3.25 [20]. Other systems treated nebulization stability as an explicit design requirement, including TG4C-LNPs formulated at a 50:10:38.5:1.5 TG4C/DOPE/cholesterol/DMG-PEG_2000_ molar ratio [43], PEG-free ionizable liposome–mRNA lipocomplexes with ordered lipid bilayers [99], and onion-like LNPs that preserved structural integrity and siRNA stability during continuous intratracheal nebulization [29].

Although quantitative aerosol reporting remains uneven, several studies provide useful benchmarks. SC12CDClickpropylamine siRNA nanocomplexes nebulized with an Aeroneb Pro produced volumetric median diameters of approximately 4 micrometers and fine particle fractions of approximately 57%, while largely retaining functional knockdown [37]. Crosslinked chitosan nanoparticles aerosolized with a Pari LC Sprint jet nebulizer-maintained encapsulation efficiencies above 96% and preserved siRNA stability; fine particle fractions were 54 ± 11% for plain chitosan and 57.3 ± 1.9% for the 10:1 chitosan/siRNA formulation [105]. pH-sensitive guanidinylated O-carboxymethyl chitosan (GOCMCS)/N-2-hydroxypropyltrimethyl ammonium chloride chitosan (N-2-HACC) nanoparticles retained siRNA stability after nebulization and achieved fine particle fractions above 60% [89]. Acoustomicrofluidic nebulization generated siRNA aerosols with tunable mean aerodynamic diameters of approximately 3 micrometers while preserving post-nebulization gene silencing [114]. These results demonstrate the feasibility of respirable siRNA aerosols while underscoring the need to report pre- and post-nebulization particle size, aggregation, RNA integrity, and function consistently.

Other systems support feasibility but provide less decisive product-development evidence because aerosol output or post-processing characterization was incomplete. Piperazine-substituted chitosan/siRNA complexes preserved size and integrity after PenCentury microsprayer aerosolization [36], while chitosan/siRNA nanoparticles pneumatically aerosolized through a nebulizing catheter showed only a modest change in in vitro silencing before and after aerosolization [91]. A1AT mRNA lipoplexes retained functional protein production after aerosolization, although transfection efficiency declined and was partly restored by increasing formulation input [16]. Such findings remain encouraging, but product robustness also requires aerosol-performance, RNA-stability, delivered-dose reproducibility, and device-compatibility data.

### 4.3. Device Effects on RNA Stability and Aerosol Performance

The inhalation device must be treated as part of the formulation system because aerosol-generator mechanics can determine whether RNA nanocarriers remain intact. In comparative studies, selected soft-mist and controlled low-shear platforms preserved mRNA formulations more effectively than conventional nebulizers. A Rayleigh-breakup soft-mist inhaler aerosolized mRNA-loaded liposomes while maintaining colloidal stability, mRNA integrity, and transfection efficiency, whereas a conventional vibrating mesh nebulizer caused substantial formulation losses [56]. In another comparison, a prefilled-syringe soft-mist inhaler preserved mRNA-LNP physicochemical properties and encapsulation efficiency, while the other tested soft-mist and mesh devices induced particle alteration or mRNA degradation [57]. An mRNA-LNP containing AX4, DSPC, cholesterol, and DMG-PEG_2000_ at a 35:16:46.5:2.5 molar ratio also performed best with a soft-mist inhaler after trehalose-buffer modification [15].

Alternative platforms reinforce the importance of device-specific testing. A microfluidic aerosolization system prevented mRNA-LNP aggregation, encapsulation loss, and morphological deformation relative to a conventional vibrating mesh nebulizer [59]. Laminar Fluid Ejection also preserved RNA-LNP integrity more effectively than a commercial nebulizer. The commercial device increased LNP size from 85 to 300 nanometers and reduced encapsulation efficiency from 100% to 39%, whereas Laminar Fluid Ejection produced reported encapsulation efficiencies of 94% for mRNA and 102% for saRNA and generated three-fold higher luciferase activity [58]. These comparisons demonstrate that advances in carrier design do not automatically translate into inhalable products unless compatibility with the intended device is verified.

Vibrating mesh nebulization produced more conditional results. Two mesh devices generated droplets of 4.80–4.89 micrometers and 3.56–3.59 micrometers, with mass median aerodynamic diameters of 4.03–4.84 micrometers but still caused lipoplex aggregation and reduced LNP encapsulation efficiency [60]. A separate study found cargo-dependent responses: siRNA-LNPs aggregated but continued to protect siRNA and retained biological activity, whereas mRNA-LNPs showed cargo degradation and reduced function [55]. These outcomes indicate that performance within one device class depends on RNA modality, lipid composition, buffer conditions, and nanoparticle architecture.

### 4.4. Aerosol Characterization and Inhalation-Platform Performance

Aerodynamic characterization provides a direct link between formulation processing and inhalable product performance. Aspherical siRNA-loaded microrods evaluated with a Next Generation Impactor achieved a mass median aerodynamic diameter (MMAD) as low as 3.37 micrometers and a geometric standard deviation (GSD) as low as 1.46 [75]. Chitosan–tripolyphosphate mRNA nanoparticles aerosolized with a vibrating mesh nebulizer and assessed by Next Generation Impactor produced reported optimal aerodynamic diameters of 1–5 micrometers [94]. Spray-freeze-dried mRNA-LNP powders achieved MMADs of 3–4 micrometers and fine particle fractions of approximately 40% [51], while freeze-dried and cryomilled saRNA-LNP powders retained approximately 60% of their original functionality and achieved an MMAD of 4.13 ± 0.26 micrometers [53]. These measurements connect manufacturing and aerosolization directly with respirable delivery potential.

Exposure-system studies add information on delivery uniformity and reproducibility. A four-port nose-only chamber coupled to a Collison nebulizer produced liposomal drug-carrier aerosols with a number-based mean particle size of approximately 130 nanometers, a mass median particle diameter of approximately 270 nanometers, an aerosol-mass coefficient of variation below 12%, and approximately 1.4% of the nebulized mass available at each port during 60 min of continuous aerosolization [116]. MRT5005 provides an important clinical distinction: it was a nebulized codon-optimized CFTR mRNA-LNP administered to adults in single-dose cohorts of 8–24 mg, five-weekly-dose cohorts of 8–20 mg, and a five-daily-dose cohort of 4 mg [19]. Although its clinical outcome is discussed elsewhere, its administration establishes clinical use of a nebulized mRNA-LNP dosage form.

### 4.5. pMDI and DPI Formulations

Portable inhalers introduce additional constraints involving propellant compatibility, powder dispersibility, aerosol performance, structural integrity, and reproducible actuation. Mannitol microparticles containing TPP-modified PAMAM dendriplexes produced fine particle fractions of 50–53% for pMDIs and 39% for DPIs in Andersen Cascade Impactor testing without impairing knockdown efficiency [49]. Hydrofluoroalkane (HFA)-based pMDIs containing PAMAM dendriplexes in mannitol and a biodegradable, water-soluble co-oligomer maintained structural integrity after particle preparation and long-term HFA exposure, with respirable fractions up to 77% [106]. Baclofen-functionalized trimethyl chitosan (Bac-TMC)/tripolyphosphate (TPP) nanoparticles containing survivin-siRNA and formulated with mannitol in a pMDI achieved a fine particle fraction of 45.39 ± 2.99% [117]. These systems demonstrate technical feasibility, but compatibility with one propellant, excipient matrix, dendriplex, or chitosan nanoparticle cannot be generalized to unrelated RNA carriers.

### 4.6. Spray-Dried and Spray-Freeze-Dried Powders

Dry-powder engineering can address inhalability and storage instability, but requires tightly controlled processing. Naked siRNA co-spray-dried with mannitol and 50% *w*/*w* L-leucine achieved an emitted fraction of approximately 80% and a fine particle fraction of 45%, while gel-retardation and high-performance liquid chromatography (HPLC) analyses indicated preserved structural integrity [50]. Powders containing more than 6% *w*/*w* siRNA used HSA as a dispersion enhancer, achieved fine particle fractions above 50%, and retained up to 78% intact siRNA with preserved bioactivity [97]. Spray-dried lipid–polymer hybrid nanoparticles with trehalose formed approximately 3-micrometer particles; encapsulation efficiency decreased from approximately 99% in fresh nanoparticles to 80% after processing, while comparable silencing was retained [81]. Spray-dried LNP-siRNA powders in 5% *w*/*v* lactose redispersed to approximately 150 nanometers, maintained residual moisture below 5%, limited RNA loss to below 15%, and retained bioactivity [62].

Spray-drying stress remains a major limitation. In EGFP-siRNA/mannitol powders, higher inlet temperatures and more intensive atomization shifted particle morphology from spherical toward irregular structures, increased agglomeration, altered mannitol polymorphism, and reduced siRNA chemical stability and transfection efficiency [110]. Spray drying is therefore conditionally useful rather than inherently stabilizing and requires control of thermal exposure, atomization, excipient crystallinity, residual moisture, and redispersibility.

Low-temperature processing is particularly relevant for mRNA. Spray-freeze-dried mRNA-LNP powders containing cryoprotecting sugars achieved MMADs of 3–4 micrometers and fine particle fractions of approximately 40%, with pulmonary delivery comparable to instilled liquid formulations [51]. A pH-modified spray-freeze-drying method improved encapsulation and transfection relative to conventional spray freeze-drying and preserved performance during storage better than liquid mRNA-LNPs, although fresh-powder activity remained below that of intact fresh-liquid formulations [111]. PEG(12)KL_4_/mRNA complexes processed by spray drying or spray freeze-drying retained aerosol properties and biological activity, producing deep-lung luciferase expression after intratracheal powder administration [107]. Dry-powder mRNA delivery is therefore feasible, but must be evaluated against both fresh-liquid potency and long-term storage performance.

### 4.7. Lyophilization, Thin-Film Freeze-Drying, and Storage Stability

Storage stability remains a major bottleneck, particularly for aqueous RNA-nanoparticle suspensions. saRNA-LNPs containing 20% *w*/*v* sucrose remained stable as liquids for 8 weeks at −80 °C and −20 °C but degraded at 4 °C and 20 °C. Freeze-drying preserved LNP integrity for 8 weeks at 4 °C, and subsequent cryomilling with 4% *w*/*w* leucine retained approximately 60% of the original functionality while producing an MMAD of 4.13 ± 0.26 micrometers [53]. OEI-HD/siRNA polyplexes were stabilized by PEG grafting or PEI–PEG mixing in isotonic saline, while Pluronic P68/F68 or Alveofact stabilized formulations in isotonic glucose; lyophilization enabled room-temperature dry storage for up to 3 months [96]. NAC in a sucrose-based lyoprotectant matrix preserved LNP physicochemical integrity and siRNA encapsulation after lyophilization [54].

TFFD illustrates how manufacturing can improve redispersibility and aerosolization simultaneously. Solid lipid nanoparticles retained size, PDI, and zeta potential after TFFD and reconstitution, whereas conventional shelf freeze-drying did not preserve these attributes. TFFD powders also outperformed spray-dried powders aerodynamically and preserved the biological function of encapsulated siRNA [52]. Pre-processing activity alone therefore does not establish product readiness; critical quality attributes must survive drying, storage, reconstitution where applicable, and aerosolization.

### 4.8. Excipient Design for Aerosol Performance and RNA Preservation

Excipients are product-enabling components that support dispersion, matrix formation, interfacial stabilization, cryo- or lyoprotection, particle-size control, redispersibility, isotonicity, and RNA recovery. Leucine, mannitol, trehalose, dextran, sucrose, lactose, triarginine, and HSA each improved at least one processing, powder, recovery, or stability attribute in specific formulations [50,53,71,83,86,97,112]. Poloxamer 188, Pluronic surfactants, Alveofact, polyvinyl alcohol, glucose, citrate buffer, and NAC-containing matrices similarly improved aerosolization stability, material recovery, isotonicity, colloidal properties, or lyophilized LNP performance [14,54,82,96]. No excipient is universally optimal; its function depends on carrier type, RNA cargo, manufacturing process, and device.

Ternary Tre:Dex/leucine matrices for siRNA-loaded lipidoid–polymer hybrid nanoparticles provide a particularly defined example. Increasing leucine from 20% to 60% *w*/*w* raised surface leucine from 39.2 to 68.1 mol% and improved flowability, while aerosol yield peaked at 30–40% *w*/*w* leucine. A formulation containing 40% leucine and a 10:90 *w*/*w* Tre:Dex ratio preserved siRNA integrity and produced homogeneous deep-lung deposition [112]. Long-term studies clarified the components’ functions: leucine formed a dispersion-enhancing shell, trehalose protected nanoparticle surfaces during manufacture and storage, and dextran promoted porosity while limiting trehalose recrystallization [113]. This evidence is especially translationally informative because it connects excipient function with aerosol performance, RNA integrity, and storage stability.

A processing-relevant PEG trade-off also emerges. Increasing PEG-lipid content improved colloidal stability during aerosolization but reduced in vitro transfection efficiency [45], while higher PEG-lipid molar ratios in another LNP study reduced particle size but also decreased intracellular mRNA expression [44]. Surface stabilization may therefore aid processing or mucus-facing stability while excessive shielding reduces cell interaction or functional delivery. PEG content should consequently be verified before and after aerosolization rather than selected solely for colloidal stability.

### 4.9. Translational Implications of Processing and Device Performance

Although inhalable RNA formulations have advanced substantially, fewer studies provide the integrated evidence required for product development: manufacturing-relevant processing, pre- and post-processing analytical characterization, aerosol metrics, storage stability, delivered-dose characterization and, where measured, reproducibility, and device-specific compatibility [11,12]. The most informative studies connect formulation design to MMAD, fine particle fraction, emitted or respirable fraction, encapsulation retention, RNA integrity, and post-processing bioactivity [37,49,50,51,75,97,106]. Additional product-relevant measures include residual moisture, redispersed particle size, post-aerosolization or post-drying activity, and stability after storage, reconstitution, or solid-state processing [52,53,62,112,113].

A practical development sequence follows from this evidence. Early candidates should retain particle size, heterogeneity, morphology, surface properties, RNA encapsulation, and RNA integrity after the intended processing step. Aerosol-stage candidates should then demonstrate reproducible droplet or particle-size distributions, respirable fraction, emitted dose or device output, and retained function after actuation. Dry powders additionally require redispersibility where applicable, moisture control, crystallinity or amorphous-state stability, and storage data under relevant conditions. Advancement should depend on device-specific evidence rather than assuming compatibility can be transferred among nebulizers, soft-mist inhalers, pMDIs, and DPIs [14,15,55,57,58,59,60]. Table 3 summarizes these readiness checkpoints.

Overall, three restrained conclusions emerge. First, inhalable RNA nanomedicines can be formulated as nebulized liquids, soft-mist aerosols, portable-inhaler products, and dry powders, but success remains strongly process- and device-dependent. Second, aerosolization and drying can preserve RNA function when supported by suitable excipients, buffers, low-damage devices, and optimized processing, but can also cause aggregation, encapsulation loss, morphological change, RNA degradation, or reduced activity when poorly matched to the carrier. Third, scientific and translational progress must remain distinct. A new carrier, publication, or preclinical efficacy result represents scientific progress; translational maturity requires the formulation, manufacturing process, device compatibility, storage stability, aerosol performance, and post-processing biological function to be characterized as an integrated product system.

## 5. Post-Deposition Lung Barrier Navigation and Cellular Access

**Figure 4:**
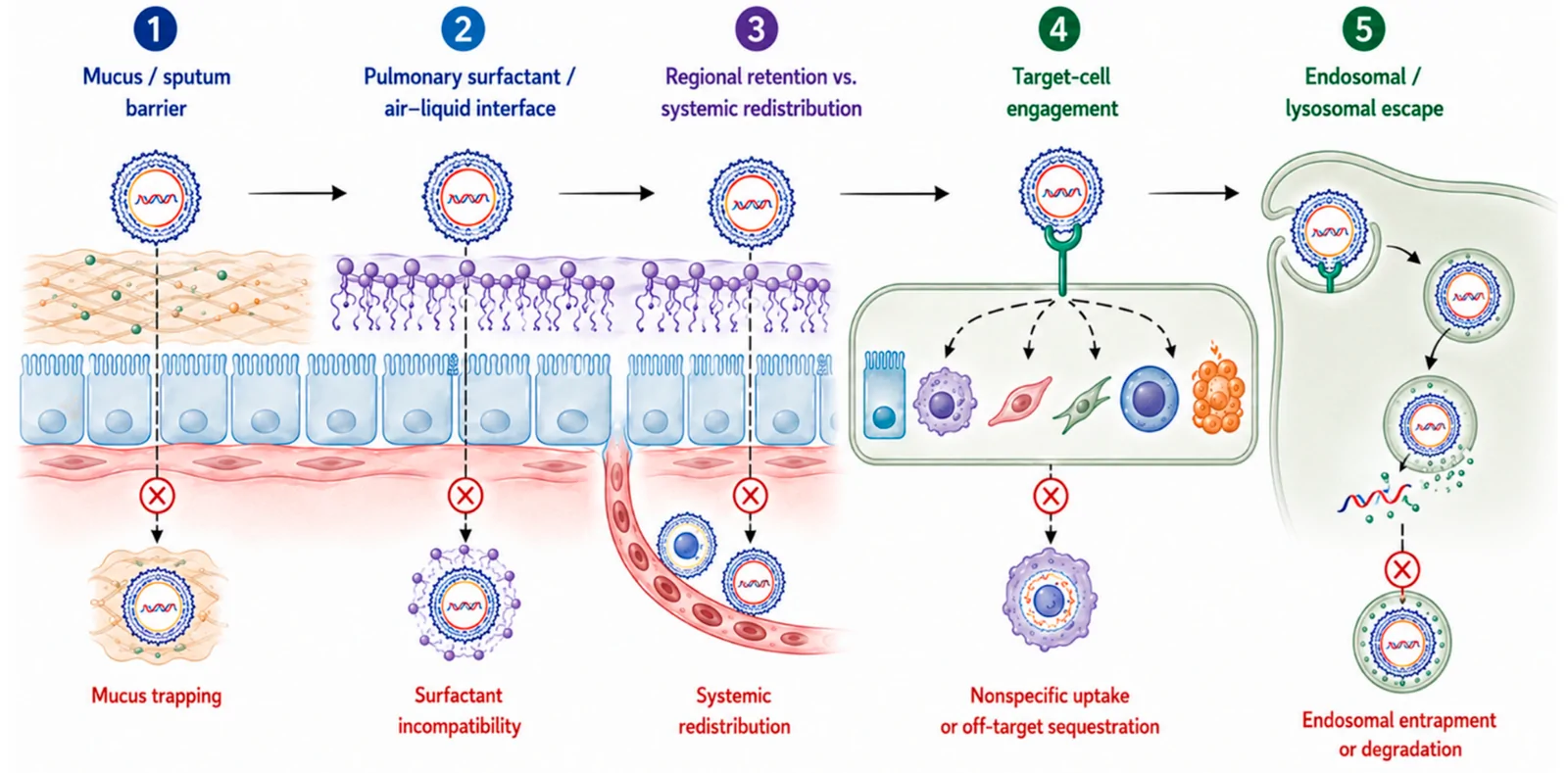
Post-deposition biological access map for inhalable RNA nanomedicines. The figure depicts the sequential barriers after aerosol deposition: mucus mobility, surfactant-interface compatibility, regional retention versus systemic escape, target-cell engagement, nonspecific uptake, endosomal escape, and endosomal entrapment. Aerosol stability and lung deposition are necessary but insufficient; meaningful pulmonary RNA delivery requires verified access across extracellular, cellular, and intracellular barriers (Prepared with the assistance of Gemini NotebookLM pro and OpenAI ChatGPT Plus).

After aerosol deposition, inhalable RNA nanomedicines must navigate several biological barriers before the RNA cargo reaches disease-relevant intracellular sites. Deposition is therefore an entry point rather than proof of pulmonary delivery. The post-deposition sequence comprises mucus or sputum traversal, compatibility with the surfactant-rich air–liquid interface, regional distribution and retention, target-cell engagement, and release beyond endosomal or lysosomal confinement (Figure 4). These steps are interdependent: a feature that improves extracellular mobility may reduce cellular uptake, while a carrier that reaches the lung may still fail to localize, persist, or release RNA in the intended cell population.

### 5.1. Extracellular Barrier Navigation: Mucus, Sputum, and Mucin-Rich Interfaces

Inhalable RNA nanomedicines must traverse mucus, sputum, mucin-rich fluids, and mucus-lined epithelium before reaching target cells, and no single surface modification resolves this barrier universally. HA- and PEG-modified lipoplexes diffused through fresh human airway mucus with Brownian-like trajectories, whereas unmodified cationic lipoplexes were trapped; HA also increased uptake by CD44-expressing A549 cells [34]. PEGylation, however, was context-dependent. In cystic-fibrosis-relevant models, PEGylated lipid–polymer hybrid nanoparticles did not improve transport through complex patient sputum, which varied with microbial colonization and mucin composition, while non-PEGylated hybrids performed better in mucus-lined air–liquid interface models [26]. This illustrates the pulmonary PEG dilemma: greater PEG content may improve shear resistance, colloidal stability, or mucus mobility [18,45], but excessive or poorly matched shielding can reduce cellular uptake, intracellular expression, or functional delivery [26,44,45].

Other systems treat mucus as a dynamic pathological barrier rather than a passive gel. INU-VS-g-(PMeOx;bAPAE)-based siRNA polyplexes remained intact at 5 mg/mL mucin and retained high muco-diffusivity [61]. Charge-reversal nanocomplexes crossed mucus while negatively charged, then reversed charge in acidic inflamed alveoli to promote alveolar-macrophage delivery [63]. NAC-containing lyophilized LNPs used extracellular mucolysis to improve penetration in mucus-hypersecretory models [54], whereas HA-DA-coated poly(β-amino ester)/siRNA systems combined charge screening, dopamine-mediated bioadhesion, CD44-mediated macrophage internalization, and endo/lysosomal escape [42]. Spray-dried LNP-siRNA powders also crossed the lung mucus layer after redispersion to approximately 150 nanometers, linking post-processing particle recovery with barrier performance [62]. Mucus penetration therefore depends on surface charge, coating composition and density, disease-specific mucus composition, particle recovery after processing, and bioresponsive behavior after deposition.

### 5.2. Pulmonary Surfactant and Air–Liquid Interface Access

Pulmonary surfactants can reshape cellular access rather than serving only as a compatibility medium. Alveofact-coated PEI polyplexes diffused through mucus in a three-dimensional air–liquid interface model and were internalized by lung epithelial cells, suggesting that surfactant coating can improve access across mucus-covered epithelium [80]. Conversely, natural pulmonary surfactant reduced internalization of siRNA-loaded dextran nanogels while preserving their gene-silencing activity, whereas the tested cationic lipid carrier, Lipofectamine RNAiMAX, was described as incompatible with pulmonary surfactant [46]. Surfactant exposure therefore cannot be assumed to be uniformly stabilizing or destabilizing.

Surfactant-protein-inspired systems provide a stronger mechanistic basis for interface engineering. SP-B-mediated delivery correlated with fusion to anionic endosomal membranes and successful cytosolic siRNA delivery [38]. SP-B-containing proteolipid-coated nanogels increased uptake and cytosolic delivery, enabled TNF-α silencing, and showed high affinity for resident alveolar macrophages [40]; preserving the functional SP-B coat through lyophilization, reconstitution, and nebulization maintained structural integrity and delivery performance in a human lung epithelial cell line [108]. Synthetic KL_4_ complexes retained siRNA-delivery activity in the commercial pulmonary surfactant Infasurf and entered cells through energy-dependent, clathrin-mediated endocytosis [65]. In human lung mucus models, DOPE- or DSPC-containing LNPs penetrated mucus but formed different protein coronas, with stability and transfection depending on the helper-lipid–RNA-cargo pairing [13]. Surfactant- and lung-interface performance should therefore be tested directly rather than inferred from buffer-based colloidal stability.

### 5.3. Regional Lung Distribution and Pulmonary Retention

Deposition, distribution, and retention are related but distinct requirements. Broad regional access was reported for piperazine-substituted chitosan/siRNA complexes distributed throughout the lung [36], chitosan/siRNA nanoparticles deposited across alveolar and bronchiolar regions after aerosolization [91], hyperbranched poly(β-amino ester) (hPBAE) mRNA polyplexes distributed across all five lung lobes and transfecting 24.6% of total lung epithelial cells after one inhaled dose [68], and TG4C LNPs producing uniform bioluminescence across all five mouse lung lobes [43]. Dry-powder mRNA-LNPs also generated reporter expression in the trachea and lungs after intratracheal administration [83]. These findings demonstrate broad access but not necessarily durable target-site exposure or cell-selective delivery.

Retention studies clarify post-deposition fate. PEI-C/PAI-1 siRNA polyplexes showed prolonged pulmonary retention after intratracheal administration [21], ODN/PLGA/PEI large porous particles persisted in rat lungs for at least 14 days [66], and local intratracheal liposomal delivery produced higher peak lung concentrations and substantially longer retention than intravenous delivery [6]. Perfluorocarbon miRNA nanocapsules were internalized by lung epithelial and tumor cells and remained in the lung beyond 48 h [64]; lipid–polymer hybrids showed longer retention than lipoplexes by single-photon emission computed tomography/computed tomography (SPECT/CT) and gamma counting [67]; and PEI-based doxorubicin/siRNA systems accumulated mainly in the lungs and remained detectable for at least 3 days [31]. Aerosolized functional polyester nanoparticles also localized to the lungs and avoided liver accumulation, unlike intravenous delivery [69]. The strongest evidence combines regional imaging or biodistribution with retention time courses rather than relying only on deposition or reporter expression.

### 5.4. Cellular Targeting in the Pulmonary Microenvironment

The field is progressing from nonspecific lung exposure toward cell-defined delivery. Epithelial access remains central. Chitosan-coated PLGA nanoplexes increased respiratory epithelial uptake relative to free antisense 2′-O-methyl RNA (OMR) in an isolated perfused and ventilated rat lung [92]; SP-B-derived peptide/siRNA complexes showed concentration-dependent uptake in A549 cells [93]; and approximately 200-nanometer SC12CDClickpropylamine nanocomplexes achieved greater siRNA association in Calu-3 bronchial epithelial cells than RNAiFect at all tested mass ratios (*p* < 0.001, *n* = 3 × 4) [37]. Additional platforms reached epithelial or airway-relevant models, including A1AT mRNA lipoplexes in human bronchial epithelial cells [16], transferrin–melittin–PEI (Tf-Mel-PEI) polyplexes producing epithelial delivery and GATA3 silencing in human precision-cut lung slices [27], chitosan–tripolyphosphate mRNA nanoparticles in A549 and BEAS-2B cells [94], SM-102-based B-1 LNPs with selective epithelial transfection over immune or endothelial cells [100], intranasal sulfonium LNPs transfecting club and ciliated cells [41], and PACE polyplexes reaching epithelial and antigen-presenting cells [5].

Pulmonary RNA delivery is also extending beyond epithelial replacement or silencing. Dendrimer–lipid derivatives targeted Tie2-expressing lung endothelial cells [7]. In a related systemically administered platform, lipopolymer–lipid hybrids delivered mRNA to lung endothelium and immune cells, including T cells [118]. Transferrin–PEI polyplexes selectively delivered siRNA to activated T cells, particularly Th2 cells, in asthmatic mouse lungs [87]. In fibrotic models, reporter mRNA-LNPs accumulated in lung tissue, generated bioluminescence from 2 to 48 h, and associated with alveolar epithelial type 2 cells and fibroblasts [9], while perfluorocarbon emulsion polyplexes showed high uptake in primary lung fibroblasts from fibrotic mice [23]. These findings broaden the field but remain largely preclinical. Human administration of aerosolized CFTR mRNA in MRT5005 demonstrated clinical progression without directly establishing mucus traversal, target-cell engagement, or intracellular RNA access in patients [19].

### 5.5. Inflammatory-Site, Macrophage, and Tumor Access

Inflammatory and tumor-associated sites require both extracellular navigation and cell-specific engagement. Several strategies support macrophage-directed delivery. Pyrrolidinium-terminated phosphorus dendriplexes were taken up more strongly by LPS-activated RAW264.7 macrophages than morpholinium-containing dendriplexes [24], PAMAM dendriplexes showed greater uptake than non-complexed siRNA [84], and neutrophil membrane-coated PLGA nanoparticles targeted inflammatory and macrophage cells at inflamed lung sites [25]. Mesoporous silica siRNA microrods were readily taken up by differentiated THP-1 macrophage-like cells, with chloroquine incorporated to promote endosomal escape [75]. PLGA microparticles transfected up to 55% of cells in THP-1 and primary-monocyte models [85], NLD2 LNPs transfected alveolar macrophages in vivo [8], and hydrogen-assisted hybrid lipid nanoparticle (HNP)/TGF-β1 siRNA delivery enhanced deposition in fibrotic lesions while inducing macrophage HGF production and lung repair [74]. Macrophage uptake is most informative when linked to barrier traversal, diseased-site accumulation, target modulation, or functional repair rather than isolated cell-culture uptake alone.

Tumor-access studies likewise extend beyond passive deposition. An inhalable liquid-core lipid nanoplatform (LLX) dispersed throughout lung airways and enabled tumor-associated macrophage-mediated relay of p53 mRNA to NSCLC cells [2]. Extracellular-vesicle-encapsulated IL-12 mRNA showed preferential cancer-cell uptake and localized delivery to primary neoplastic lesions [39]. Star-polymer siRNA nanoparticles entered lung cancer cells, escaped the endo-lysosomal pathway, accumulated in the lungs after nebulization, and silenced βIII-tubulin and PLK1 in mouse lung tumors [33]. Perfluorocarbon miRNA nanocapsules bypassed mucus barriers, avoided immune clearance, and targeted lung epithelial and tumor cells; after one aerosolized dose, they entered more than 60% of cancer cells and most type II alveolar and bronchial epithelial cells and remained in the lung beyond 48 h [64]. Cationic dioleoyl-sn-glycero-3-ethylphosphocholine/cholesterol (ECL) nanoliposomes increased in vivo pulmonary delivery of fluorescent siRNA 26.2-fold relative to naked siRNA [30], while dual-targeted cationic lipid–HA mRNA nanoparticles accumulated mainly in lung-tumor cells and inflammatory macrophages after aerosol inhalation [10]. These results provide compelling preclinical access evidence but remain model-specific and do not establish clinical targeting.

### 5.6. Intracellular Uptake and Endosomal Access

Cellular uptake does not ensure functional RNA availability; intracellular escape and cargo release must also be demonstrated or mechanistically supported. DLin-MC3-DMA-based siRNA-LNPs promoted uptake and endosomal escape in vitro [20]. GOCMCS/N-2-HACC/siRNA nanoparticles, measuring 150–180 nanometers with a zeta potential of approximately −17 millivolts, entered A549 cells through energy-dependent endocytosis and escaped endosomes through guanidine-mediated membrane penetration and pH-sensitive swelling [89]. Other studies linked targeted uptake or cytosolic transport to RNA activity. Bac-TMC/tripolyphosphate nanoparticles increased survivin-siRNA uptake through specific gamma-aminobutyric acid type B [GABA(B)] receptor interactions [117], SAR(6)EW/TNF-α siRNA complexes transported siRNA into the cytoplasm [71], and four-in-one lipidic hyperbranched poly(β-amino ester) (O-LhPAE) polyplexes promoted uptake and endosomal escape, with the lead candidate achieving up to 93.1% mRNA transfection across 11 cell types [73]. Fast-degrading polyester nanoparticles, measuring 150–200 nanometers with a +15 to +20 millivolt zeta potential, disassembled within 4 h in phosphate-buffered saline (PBS) and produced 80–90% reporter-gene knockdown, supporting rapid carrier degradation and siRNA release [90].

PEG architecture and adjuvant selection further influence intracellular access. N-terminal PEGylation of a pH-responsive peptide with a 12-monomer PEG chain improved pulmonary mRNA delivery relative to the non-PEGylated carrier, whereas longer PEG chains reduced uptake and transfection [48]. PEGylated KL_4_ variants formed nanosized siRNA complexes and transfected A549, Calu-3, and BEAS-2B cells efficiently, but longer chains weakened siRNA–peptide interactions; PEG(12)KL_4_ was optimal within that series [95]. Among cationic amphiphilic β_2_ agonists, salmeterol enhanced siRNA delivery in epithelial and macrophage cell lines with both uncoated and Curosurf-coated nanogels, indacaterol enhanced delivery only in epithelial cells, and salbutamol and formoterol produced no enhancement, consistent with different capacities to induce lysosomal membrane permeabilization [109]. Mucus traversal, uptake, and intracellular escape must therefore be assessed together because improvement at one barrier may impair another.

### 5.7. Implications for Nanocarrier Engineering and Translation

The evidence supports a sequential post-deposition model: carriers must remain structurally and chemically fit after processing, cross mucus or surfactant-rich interfaces, distribute or persist in relevant regions, engage disease-relevant cells, and release RNA beyond endosomal or lysosomal restriction [18,34,42,46,63,64,67]. The principal relationships between these access barriers, nanocarrier engineering strategies, and cellular targets are summarized in Table 4. Formulation innovation and preclinical mechanistic evidence are substantial, but translational maturity remains uneven. Many studies demonstrate new chemistries, pulmonary deposition, reporter expression, or preclinical activity; fewer establish manufacturing-relevant process control, reproducible device compatibility, performance in human disease barriers, or clinical target-cell delivery [11,12]. The clinical aerosolized CFTR mRNA trial established human administration of an inhaled RNA-LNP but did not directly report the mucus traversal, target-cell engagement, or intracellular-access measurements emphasized in preclinical studies [19].

Post-deposition claims should therefore be anchored across the formulation–aerosolization–delivery sequence. Before and after nebulization, spray drying, spray freeze-drying, lyophilization, or redispersion, carriers should be evaluated for particle size, heterogeneity, aggregation, morphology, RNA integrity, encapsulation, surface charge, coating integrity, and formulation stability because processing can change the material delivered to the lung [14,52,54,56,59,62,111]. Barrier-specific verification should then use multiple-particle tracking, diffusion profiling, small-angle X-ray scattering (SAXS), human mucus or sputum, mucin-rich media, surfactant exposure, and air–liquid interface cultures for extracellular barriers [13,26,34,46,61,62,80]; in vivo imaging system (IVIS), SPECT/CT, gamma counting, and regional imaging for distribution and retention [31,36,43,64,67]; and flow cytometry, confocal microscopy, precision-cut lung slices, co-culture systems, and in vivo biodistribution for cellular access [7,27,35,64,80,87]. Each method has limitations: simplified mucus may miss patient-specific sputum heterogeneity [26], cell lines may not reproduce in vivo uptake, imaging may show localization without intracellular release, and reporter expression may combine deposition, uptake, escape, and translation. Strong evidence therefore requires convergence across orthogonal assays rather than one favorable readout.

Pulmonary immunotoxicity is inseparable from access even when efficacy is considered separately. Cationic carrier chemistry can cause inflammation or immune responses, some inhaled formulations induce dose-dependent cytokine release, and surfactant interactions or macrophage uptake can alter compatibility and local behavior [46,47,94]. Design variables that should be assessed alongside delivery include biodegradable materials, controlled or reversibly shielded charge, PEG or biomimetic coatings, dose optimization, charge-reversal or coating-shedding systems, lysosomal-delivery adjuvants, mucolytic lyoprotectant matrices, and reactive-species-scavenging dopamine-containing HA coatings [18,42,47,54,63,73,109]. A formulation should advance only when it preserves analytical quality after processing, crosses the relevant barrier, reaches the intended cell population, demonstrates functional intracellular RNA access, and avoids unacceptable delivery-related inflammation.

## 6. Pharmacodynamic Activity, Safety, Benchmarking, and Translational Interpretation

### 6.1. Scope of Pharmacodynamic Evidence

**Figure 5:**
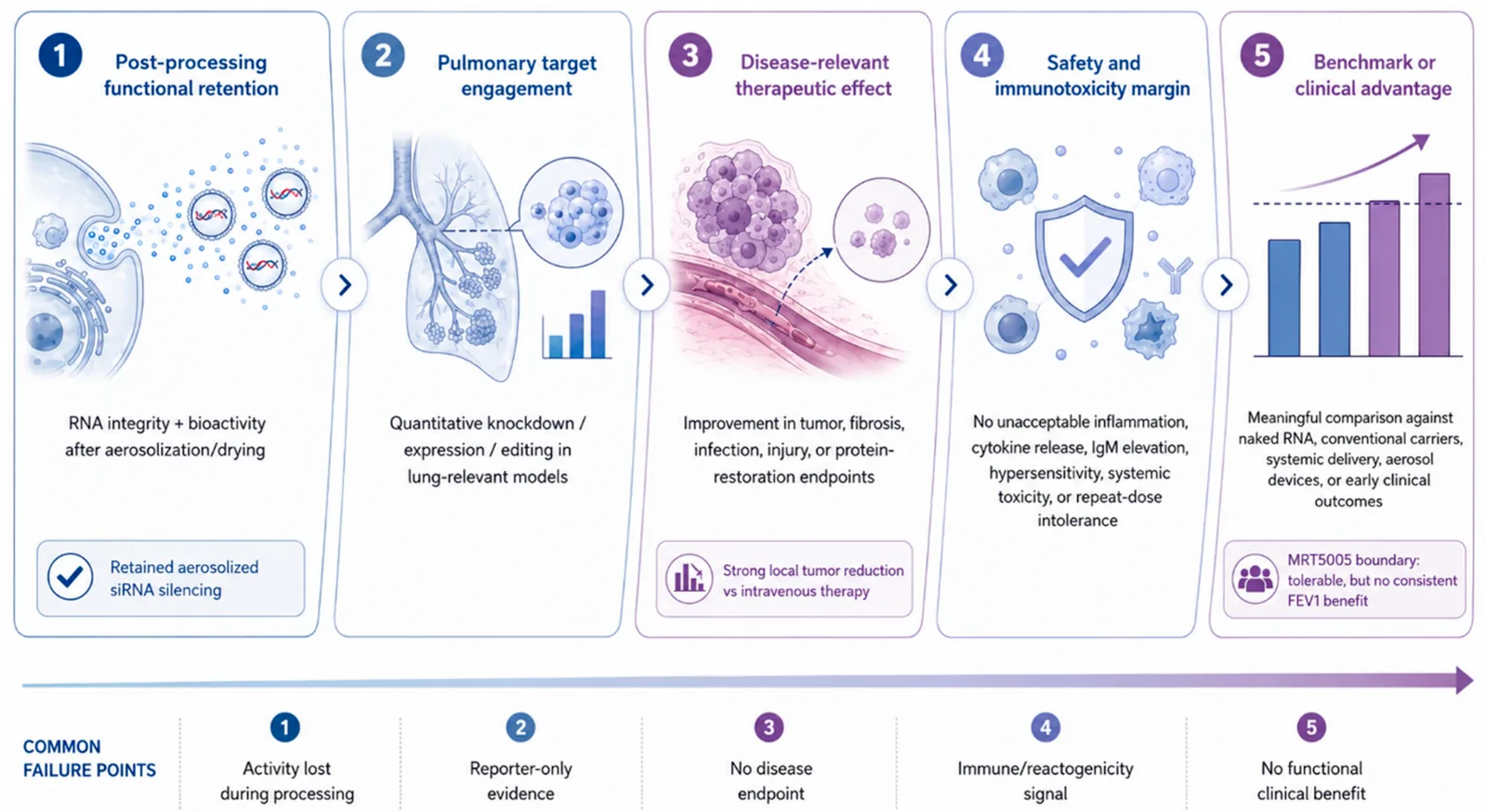
From pulmonary RNA activity to translational confidence. The figure presents five evidence gates: functional retention after aerosolization or drying, quantitative target engagement, disease-relevant therapeutic effect, an acceptable safety margin, and benchmark or clinical advantage. Common failure points include processing-related activity loss, reporter-only evidence, absence of a disease endpoint, reactogenicity or immunotoxicity, and lack of clinical benefit (Prepared with the assistance of Gemini NotebookLM pro and OpenAI ChatGPT Plus).

The pharmacodynamic literature on inhalable RNA nanomedicines has progressed from proof of delivery to measurable biological activity in pulmonary cells, ex vivo lung tissues, and disease models. Reported effects include reporter-gene silencing, therapeutic target knockdown, mRNA-derived protein expression, cytokine modulation, antiviral activity, antitumor effects, fibrosis suppression, and selected genome-editing signals [1,4,17,20,25,28,47]. Additional disease-relevant activity includes GM-CSF-mediated surfactant clearance, HGF- or Tbx2-associated lung repair, influenza-antibody protection, restoration of inhaled CFTR expression, and ex vivo GAPDH silencing in human precision-cut lung slices [3,8,18,43,62,73]. The evidence nevertheless remains uneven. Many studies show that RNA can remain structurally intact or biologically active after aerosolization or drying [50,51,52,53,97,111,112], whereas durable therapeutic benefit, clinically relevant endpoints, repeated-dose safety, and human deployment are less consistently established [11,12,19]. MRT5005 is an important clinical example of aerosolized CFTR mRNA-LNP administration, but it produced no beneficial or consistent FEV_1_ effect [19]. Figure 5 summarizes this evidence-gated distinction between pulmonary activity and translational confidence.

For siRNA systems, the most informative pharmacodynamic signals quantify knockdown in pulmonary-delivery formulations and relevant biological models. Representative effects include greater than 60% EGFP knockdown with PEI-based nanocomplexes after intratracheal administration [47], 40–80% in vitro silencing with piperazine-substituted chitosan complexes [36], a 45% TNF-α reduction over 48 h with sustained knockdown for up to 72 h in monocyte models [85], 80–90% luciferase knockdown after nebulization using only 5 picomoles of siRNA [90], a 68% in vivo EGFP reduction after aerosolized chitosan/siRNA administration [91], 88% GATA3 silencing in human precision-cut lung slices [27], and greater than 90% protein downregulation in vitro with up to 50% GAPDH silencing in human precision-cut lung slices without increased cytokine levels [62]. Pulmonary RNAi can therefore be potent, but potency depends on carrier architecture, aerosolization tolerance, target-cell access, and model stringency rather than the RNA cargo alone.

mRNA systems show a parallel but distinct maturity pattern. Localized protein expression includes functional A1AT capable of inhibiting trypsin and elastase [16], detectable DNAI1 protein in nonhuman-primate lungs at exposures overlapping those that rescued ciliary function in an in vitro primary ciliary dyskinesia (PCD) model [17], restored pulmonary CFTR expression in a deficient animal model [18], GM-CSF-mediated surfactant clearance in aPAP [8], and Tbx2 expression with lung-function restoration in silicosis mice [73]. hPBAE polyplexes generated 101.2 nanograms per gram luciferase protein 24 h after inhalation, transfected 24.6% of total lung epithelial cells after one dose, and maintained consistent expression after repeated dosing without local or systemic toxicity [68]. These studies establish biological feasibility while preserving an important distinction: reporter expression confirms delivery competence, whereas disease-relevant protein restoration or functional rescue provides stronger translational support.

### 6.2. Disease-Relevant Therapeutic Activity

The most persuasive pharmacodynamic evidence links RNA-mediated molecular effects to disease modification. In inflammatory and ALI models, neutrophil membrane-coated TLR4 siRNA nanoparticles reduced TNF-α and IL-1β, downregulated TLR4, TRAF6, XIAP, and NF-κB, increased aquaporin 1 (AQP1) and aquaporin 5 (AQP5), and attenuated ALI without normal-tissue toxicity [25]. Anti-miR-155 AMO155c-R3V6-GA micelles increased suppressor of cytokine signaling 1 (SOCS1) expression and decreased pro-inflammatory cytokines [70], while RDDsT TNF-α siRNA nanocomplexes reduced pro-inflammatory factors and promoted pulmonary-function recovery [63]. SAR(6)EW/TNF-α siRNA dry powder ameliorated ALI [71], and HA-DA-coated poly(β-amino ester)/siRNA combined TNF-α regulation with reactive oxygen species (ROS)- and reactive nitrogen species-scavenging to protect lung tissue, attenuate inflammatory cytokine responses, and ameliorate LPS-induced injury [42]. Anti-inflammatory RNA therapy is therefore most informative when cytokine modulation is paired with histological, functional, or injury-resolution endpoints.

Fibrosis and chronic lung disease studies provide similarly important signals. Inhaled TGF-β1 siRNA-LNPs silenced TGF-β1 mRNA, inhibited epithelial–mesenchymal transition, reduced inflammatory infiltration and extracellular-matrix deposition, and protected lung tissue from bleomycin toxicity without systemic toxicity [20]. PEI-C/PAI-1 siRNA polyplexes downregulated PAI-1 and reduced collagen deposition [21], while perfluorocarbon emulsion polyplexes combining CXCR4 inhibition with PAI-1 silencing were effective in ALI and early fibrogenic IPF and significantly increased survival in late-stage IPF [23]. Inhaled HP08LOOP LNPs enabled IL-11 scFv secretion and inhibited pulmonary fibrosis more effectively than inhaled or intravenously injected IL-11 scFv in suppressing fibroblast activation and extracellular-matrix deposition [72]. In aPAP, nebulized GM-CSF mRNA promoted macrophage-mediated surfactant clearance and reduced surfactant-protein accumulation more effectively than recombinant GM-CSF [8]. These findings support a mechanistic rationale for pulmonary RNA therapy in fibrotic and protein-deficiency diseases, although long-term remodeling durability, repeat-dose immunotoxicity, and clinical reversibility remain insufficiently established.

Cancer studies provide strong preclinical examples of local pulmonary delivery outperforming systemic treatment. Inhaled polymer-assisted compaction of DNA (pacDNA) suppressed KRAS expression, inhibited orthotopic lung-tumor growth, and required substantially lower dosing than intravenous pacDNA [1]. Intratracheal liposomal delivery increased lung retention and improved anticancer efficacy relative to intravenous administration [6]. Inhaled drug and RNA-resistance-suppression therapy produced high antitumor activity with low adverse effects [79], while a related inhalation-exposure study reduced tumor volume by more than 90%, compared with approximately 40% after intravenous doxorubicin [116]. PFC-based miR-34a nanocapsules suppressed metastatic outgrowth, stimulated antitumor immunity, produced negligible cytokine release, and doubled median survival relative to paclitaxel chemotherapy [64]. Additional activity was reported with p53 mRNA LLX, IL-12 mRNA extracellular vesicles, βIII-tubulin/PLK1 siRNA star nanoparticles, KRAS-directed CRISPR LNPs, Mcl-1 siRNA nanoliposomes, and Bcl-2 siRNA/doxorubicin co-delivery [2,4,30,31,33,39], while aerosolized functional polyester nanoparticles achieved lung-localized silencing in orthotopic tumors [69]. The consistency of local antitumor benefit is notable, but remains preclinical and establishes proof of concept rather than clinical efficacy.

Disease-relevant activity also appears in infection and vaccination models. siR-7-EM/KK-46 reduced SARS-CoV-2 lung inflammation and viral titers in hamsters [28]; α_5_β_1_ siRNA-LNPs reduced *Staphylococcus aureus* pneumonia severity and bacterial burden while preventing systemic dissemination [29]; PACE mRNA vaccination induced cellular and humoral immunity that protected mice from lethal SARS-CoV-2 challenge [5]; and optimized inhaled LNPs encoding a broadly neutralizing antibody completely protected mice from lethal H1N1 challenge while delivering mRNA more efficiently than systemic LNPs [3]. These results support airway-localized RNA therapy against respiratory pathogens, although most evidence remains limited to animal models.

### 6.3. Safety and Tolerability

Safety findings are encouraging but formulation-specific and cannot be generalized across carrier classes. Several platforms reported low cytotoxicity, preserved viability, local tolerability, no normal-tissue or systemic toxicity, no chronic cytokine increase, or no observable adverse effects after high-dose or repeated administration [7,20,25,36,59,61,92]. Other well-tolerated systems include sulfonium LNPs, repeatedly dosed hPBAE mRNA polyplexes, KL_4_ or PEG-KL_4_ peptides, crosslinked chitosan nanoparticles, and PFC-based miR-34a nanocapsules [41,64,65,68,95,105,107]. Relevant examples include no adverse effects after repeated piperazine-substituted chitosan administration [36], preserved lung physiology after aerosolized OMR nanoplexes [92], no systemic toxicity with TGF-β1 siRNA-LNPs [20], no chronic cytokine increase, weight loss, or adverse high-dose toxicity with dendrimer–lipid derivatives [7], no sustained inflammation or tissue damage after sulfonium LNP delivery [41], and repeated hPBAE mRNA dosing without local or systemic toxicity [68].

The same evidence identifies important liabilities. PEG-modified PEI reduced cytotoxicity but caused severe pulmonary inflammation and elevated bronchoalveolar immunoglobulin M (IgM) [47]. Intratracheal lipoplex-siRNA caused inflammation, whereas intravenous lipoplex-siRNA reduced vascular endothelial cadherin (VE-cadherin) mRNA without cellular influx [119]. Cyclodextrin nanocomplexes retained knockdown after nebulization but were toxic at mass ratios of 50–100 [37], and chitosan–tripolyphosphate mRNA nanoparticles induced dose-dependent cytokine release [94]. Reduced in vitro cytotoxicity therefore does not necessarily predict pulmonary tolerability, and local immune activation may emerge despite efficient delivery.

Pulmonary immunotoxicity remains less thoroughly characterized than efficacy. Common assessments include viability, cytokine release, bronchoalveolar inflammatory cells, IgM elevation, histology, weight loss, gross toxicity, and limited cytokine or tolerability readouts [7,19,20,25,47,94,119]. Other studies report no local or systemic toxicity, negligible cytokine release, low inflammatory risk, or no cytokine elevation in selected preclinical or ex vivo settings [62,64,68,95]. Fewer address the broader repeated-dose profile, including innate immune activation, macrophage and neutrophil recruitment, complement-related responses, oxidative stress, and adaptive mucosal immunity. Supported mitigation concepts include biodegradable or less-cationic materials, negatively charged or shielded surfaces, optimized PEGylation, charge-reversal systems, and ROS-scavenging groups [7,18,20,41,42,63,68]. These strategies remain platform-specific and require direct verification rather than class-wide safety assumptions.

The Phase 1/2 MRT5005 trial defines the clearest clinical boundary. Among 42 adults with cystic fibrosis—31 receiving aerosolized CFTR mRNA-LNPs and 11 receiving a placebo—MRT5005 was generally safe and well-tolerated. Nevertheless, 14 febrile reactions occurred in 10 treated participants, two participants discontinued treatment, two hypersensitivity reactions occurred, cough and headache were common, and no beneficial or consistent FEV_1_ effect was observed despite stable lung function [19]. The trial therefore demonstrates first-in-human feasibility without consistent clinical benefit.

### 6.4. Benchmarking and Aerosolization-Dependent Performance

Benchmarking across carriers, routes, devices, and RNA formats is essential because pharmacodynamic performance reflects the integrated carrier–cargo–device system. Local pulmonary delivery was therapeutically distinct from systemic administration in several studies. Inhaled pacDNA inhibited lung-tumor growth at a substantially lower dose than intravenous pacDNA [1], and intratracheal liposomes improved lung retention and anticancer efficacy relative to intravenous delivery [6]. Inhaled doxorubicin with resistance-suppressing oligonucleotides produced substantially greater tumor reduction than intravenous doxorubicin [116]. Optimized inhaled LNPs encoding a broadly neutralizing influenza antibody delivered mRNA more efficiently than systemic LNPs and completely protected mice from lethal H1N1 challenge [3], while aerosolized functional polyester nanoparticles avoided liver accumulation and enabled lung-localized tumor-gene silencing relative to intravenous administration [69]. Pulmonary delivery is therefore justified when local exposure improves efficacy or reduces off-target distribution, and the formulation survives aerosolization to reach relevant cells.

Carrier comparisons show that nanocarrier engineering determines efficacy and tolerability. Non-PEGylated hybrid nanoparticles outperformed PEGylated counterparts in complex cystic fibrosis sputum and mucus-lined air–liquid interface models [26]. HA-coated liposome–protamine–DNA (LPD) particles reduced cytotoxicity and improved Bcl-2 silencing relative to uncoated LPD particles [78]. Dextran nanogels retained silencing after pulmonary-surfactant exposure, whereas the tested cationic lipid carrier Lipofectamine RNAiMAX was incompatible with surfactant [46]. LNPs achieved up to 93% firefly luciferase (Fluc) knockdown after nebulization compared with up to 30% for PEGylated lipoplexes [60], and lipid–polymer hybrids prolonged EGFP silencing while reducing acute inflammatory responses relative to cationic lipoplexes [67]. Formulation changes should thus be evaluated through coupled functional and safety endpoints, not physicochemical stability alone.

The pulmonary PEG dilemma illustrates this requirement. PEG or PEG-like shielding may improve colloidal stability, shear resistance, mucus interaction, or biocompatibility, but excessive or poorly tuned shielding may reduce cellular association, uptake, intracellular expression, or transfection [18,26,45,48]. Increasing PEG-lipid content improved aerosolization stability but reduced in vitro transfection [45], whereas an optimized PEGylated peptide assembly improved pulmonary mRNA delivery and safety relative to the non-PEGylated peptide and Lipofectamine 2000 [48]. PEGylation did not improve transport through complex cystic fibrosis sputum, where non-PEGylated hybrids were more promising [26]. PEG density, chain length, and formulation context should therefore be linked experimentally to mucus transport, post-aerosolization integrity, cell entry, intracellular delivery, and pharmacodynamic output. Reversible shielding, charge-reversal systems, and bioresponsive interfaces align conceptually with this problem, but their value must be established through direct functional benchmarking [18,45,48,63,74].

Device and processing effects are equally decisive. Selected soft-mist inhalers, laminar fluid ejection, and microfluidic aerosolization preserved mRNA-LNP or RNA-LNP integrity and function more effectively than conventional nebulization in several comparisons [56,57,58,59]. Laminar fluid ejection produced three-fold higher luciferase activity than a commercial nebulizer [58], while cyclodextrin nanocomplexes largely retained knockdown after nebulization: 39.8 ± 3.6% before and 35.6 ± 4.55% after aerosolization at a mass ratio of 20 [37]. Conversely, A1AT mRNA aerosolization reduced transfection efficiency [16], mesh nebulization preserved siRNA-LNP function but diminished mRNA-LNP activity through cargo degradation [55], and spray-drying thermal or atomization stress impaired siRNA chemical stability and transfection [110]. Leucine- or albumin-assisted spray drying, cryoprotective or lyoprotective matrices, optimized drying, buffer modification, and surfactant-compatible coatings preserved bioactivity in selected systems [50,53,54,82,97,108,111]. Ternary excipient matrices, TFFD, and spray-dried LNP-siRNA powders also supported retained activity, redispersibility, aerosol performance, or RNA preservation [52,62,112,113]. Aerosolization stability must therefore be verified analytically and biologically: particle size, heterogeneity, aggregation, morphology, RNA integrity, encapsulation, surface properties, and formulation stability before and after processing should be interpreted alongside retained knockdown, protein expression, or therapeutic effects [52,53,55,57,58,82,111].

### 6.5. Translational Interpretation

Collectively, inhalable RNA nanomedicines show pharmacodynamic activity and therapeutic potential across cancer, fibrosis, ALI, asthma, infection, genetic lung disease, pulmonary proteinosis, and silicosis [11,12]. Disease-modifying signals include fibrosis suppression, cytokine reduction, pulmonary-function recovery, cell-based ciliary rescue associated with DNAI1 protein expression in nonhuman-primate lungs, disease-corrective protein activity, and protection from lethal viral challenge [3,8,17,20,25,72]. Additional signals include tumor regression, survival extension, mutation-directed genome editing, and silicosis-associated lung-function restoration [1,2,4,64,73,116]. At the enabling end of the spectrum, studies showing RNA stability, aerosol compatibility, carrier feasibility, or reporter expression without disease modification, long-term safety, or clinical validation remain valuable but should not be treated as therapeutic validation [11,12,15,50,57,112,113].

Table 5 summarizes the principal benchmarks. A translational roadmap should be evidence-gated rather than publication-count-based [11,12]. Early maturity requires reproducible nanoparticle formation, preserved RNA integrity, acceptable encapsulation, controlled aggregation, and retained function after manufacturing or aerosolization. Intermediate maturity requires pharmacodynamic activity in relevant lung cells, air–liquid interface cultures, mucus- or surfactant-exposed systems, ex vivo human lung tissue, or disease-relevant animal models. Advanced preclinical maturity requires disease modification, repeated-dose tolerability, defined immunotoxicity, manufacturing-relevant process control, device compatibility, and benchmarking against clinically relevant alternatives. Clinical maturity requires controlled human evidence of safety and tolerability together with target engagement or meaningful functional benefit. By this standard, scientific progress in carriers, formulations, processing, and preclinical efficacy exceeds translational progress; MRT5005 remains the principal clinical example in the reviewed corpus and demonstrated feasibility without a beneficial or consistent FEV_1_ effect [19].

The central question is therefore not merely whether RNA nanomedicines can be aerosolized, but whether a formulation maintains RNA integrity, nanoparticle stability, pulmonary bioavailability, cell-specific pharmacodynamics, and acceptable immunotoxicity under clinically relevant manufacturing, storage, and delivery conditions [14,19,52,57,59,62]. Go/no-go criteria should require retained function after processing, reproducible target engagement, therapeutic effects in disease-relevant models, absence of unacceptable pulmonary inflammation or systemic toxicity, intended-device compatibility, and a credible manufacturing scale-up rationale. Formulations that improve particle properties without preserving RNA activity or disease-relevant efficacy remain scientifically informative but are not translationally advanced.

## 7. Limitations and Future Perspectives

The evidence base is limited by heterogeneity in study design, reporting, and maturity. Studies span multiple RNA modalities, carrier chemistries, pulmonary administration routes, devices, disease models, analytical methods, and endpoints, complicating direct comparison. Many demonstrate carrier formation or biological activity but incompletely or inconsistently characterize particle heterogeneity, morphology, surface properties, RNA encapsulation and integrity, redispersibility, storage stability, emitted dose, or post-aerosolization function [11,12]. Stronger reports connect carrier composition to processing-related attributes such as size, PDI, morphology, encapsulation, RNA integrity, redispersion, aerosol metrics, and retained activity [20,37,50,51,53,81,97]. Solid-state and process-focused studies further show the value of measuring storage stability, residual moisture, crystallinity, redispersibility, and post-processing bioactivity [52,54,62,110,112,113]. Future studies should harmonize critical quality attribute reporting across pre-processing, post-processing, aerosolization, redispersion, and biological testing. Without this analytical continuity, it remains uncertain whether the formulation evaluated in vitro is the formulation delivered to the lung.

Beyond analytical characterization, a second limitation is the field’s continued reliance on reporter systems, simplified cell models, and short-term endpoints. Reporter mRNA expression, GFP/EGFP silencing, luciferase activity, GAPDH knockdown, and model splicing assays are valuable for establishing delivery competence and intracellular functionality, but they do not by themselves demonstrate disease modification or clinical feasibility [1,47,56,61,62,68,90]. Similarly, commonly used A549, BEAS-2B, Calu-3, RAW264.7, and related models may not adequately reproduce disease-specific barrier properties, multicellular interactions, or in vivo patterns of cellular uptake. Future development should therefore incorporate more human-relevant systems, including mucus- and sputum-based assays, air–liquid interface cultures, precision-cut lung slices, primary epithelial and immune-cell models, diseased human samples, and, where appropriate, large-animal or clinically aligned exposure models [13,17,26,27,34,62,86]. These systems should distinguish deposition and cellular uptake from intracellular RNA release, target engagement, functional rescue, and therapeutic activity in the intended pulmonary cell compartment.

A third limitation is device and process specificity. Although several studies show preserved RNA function after aerosolization, others report aggregation, encapsulation loss, morphological deformation, cargo degradation, or reduced expression after nebulization or drying [16,55,56,57,58,59,110]. Storage and low-temperature or solid-state studies likewise show dependence on processing conditions, excipient selection, and retained function after storage, reconstitution, or redispersion [52,53,54,108,111,112,113]. Device compatibility therefore cannot be assumed across nebulizers, soft-mist inhalers, pMDI products, DPI products, microsprayers, or experimental aerosol platforms. Formulation and device should be co-optimized from the outset, with go/no-go criteria linked to emitted dose or device output, aerodynamic diameter, FPF, delivered-dose reproducibility where measured, carrier integrity after actuation, retained RNA activity, and compatibility with clinically realistic handling and storage [15,49,51,53,58,75,106]. Clinical-device alignment is equally important, as shown by MRT5005, a nebulized mRNA-LNP product tested in adults with cystic fibrosis [19].

Safety remains a major translational bottleneck. Many platforms report low cytotoxicity, preserved viability, limited histological toxicity, or favorable repeat-dose tolerability, but pulmonary immunotoxicity is not assessed consistently or in sufficient depth [7,20,25,36,41,61,92]. Other peptides, chitosan, perfluorocarbon, repeated-dose, or ex vivo systems also show favorable tolerability, although these findings remain formulation-specific rather than class-wide [48,62,64,65,68,105]. Important liabilities remain: PEG-modified PEI caused severe pulmonary inflammation and bronchoalveolar IgM elevation, cyclodextrin nanocomplexes were toxic at higher mass ratios, chitosan–tripolyphosphate mRNA nanoparticles induced dose-dependent cytokine release, and MRT5005 caused febrile and hypersensitivity reactions in participants [19,37,47,94]. Future studies should extend beyond viability and short-term histology to integrated repeat-dose immunotoxicity assessment, potentially including innate immune activation, macrophage and neutrophil recruitment, complement-related responses, oxidative stress, adaptive mucosal immunity, bronchoalveolar biomarkers, systemic cytokines, and recovery after dosing. Safety should be evaluated alongside efficacy and exposure, not added only after delivery optimization.

Future progress requires a shift from platform enthusiasm to evidence-gated product development. The strongest candidates will begin with a defined pulmonary disease mechanism and target cell, then align the RNA cargo, carrier architecture, excipient matrix, manufacturing process, aerosol device, and pharmacodynamic endpoint with that product profile. For disease-corrective mRNA, priorities include durable but controllable protein expression, repeat-dose tolerability, target-cell specificity, and functional rescue in disease-relevant models [8,17,18,19,68,73]. For RNAi and oligonucleotide systems, priorities include pathway-selective knockdown, barrier-aware delivery, intracellular bioavailability, and evidence that molecular silencing produces histological, physiological, infectious, fibrotic, or oncologic benefit [1,20,21,25,27,28,29]. A related systemically administered anti-miR-145 program in pulmonary hypertension [22], together with inhaled oligonucleotide programs in fibrosis, ALI, and oncology [23,42,64,71], further supports linking knockdown to disease modification rather than molecular activity alone. For vaccines and encoded antibodies, development should connect mucosal deposition, antigen or antibody expression, immune quality, durability, and protection within the same pathway [3,5,53].

Ultimately, translational benchmarks must distinguish scientific feasibility from product readiness. A publication, novel material, or preclinical proof of concept should not be considered translational progress unless it demonstrates adequate RNA and formulation purity, including control of process-related impurities, degradation products, residual solvents, and other contaminants; preserved RNA integrity after processing; reproducible aerosol performance; post-deposition barrier traversal; target-cell engagement; intracellular RNA function; disease-relevant pharmacodynamics; acceptable repeat-dose safety; manufacturing-relevant scale-up logic; and alignment with the intended clinical device [11,12,19]. Because purity directly influences product consistency, biological activity, safety, and regulatory acceptability, it should be evaluated using well-defined specifications and validated analytical methods throughout production, scale-up, and quality control. Applying this framework would help move inhalable RNA nanomedicines from compelling experimental systems toward clinically credible pulmonary therapeutics.

## 8. Conclusions

Inhalable RNA nanomedicines have reached a stage at which basic pulmonary-delivery feasibility is no longer the central challenge; the field now requires integrated product development. Therapeutic success depends on aligning the RNA modality with a clinically meaningful pulmonary target, preserving cargo and nanocarrier integrity through manufacturing and aerosolization, navigating disease-relevant lung barriers, reaching the intended cells, releasing functional RNA intracellularly, and achieving durable pharmacodynamic benefit without unacceptable local or systemic toxicity. Although the evidence shows substantial innovation across cargoes, carriers, dosage forms, devices, and disease models, translational maturity remains uneven. The next generation of inhalable RNA therapeutics should therefore be developed as analytically defined, device-compatible, barrier-aware, and outcome-gated products that move beyond pulmonary deposition toward clinically meaningful therapies for respiratory disease.

### Translational Roadmap and Practice Interpretation

This roadmap (Table 6) translates the review’s evidence base into practical interpretive guidance for future development. Rather than recapitulating individual carriers, RNA modalities, disease models, or section-specific findings, it identifies what can reasonably be concluded now, what should remain provisional, and what priorities are needed to move inhalable RNA nanomedicines from experimental feasibility toward responsible translational application. The central position is that pulmonary deposition alone is insufficient; translational credibility requires preserved RNA and carrier integrity, verified pulmonary access, disease-relevant pharmacodynamics, and acceptable safety within the same product-development pathway.

## Figures and Tables

**Table 1:** Therapeutic and biological target-product profiles across inhalable RNA nanomedicines.

Therapeutic and Biological Target-Product Profile	RNA Modality and Biological Target	Intended Biological Effect	Model or Translational Signal	References
Disease-corrective protein expression	mRNA encoding CFTR, DNAI1, A1AT, or GM-CSF	Restore or replace a deficient pulmonary protein	Includes CFTR-deficient models, nonhuman-primate DNAI1 delivery, alveolar-macrophage targeting in autoimmune pulmonary alveolar proteinosis (aPAP), and a Phase 1/2 CFTR mRNA trial in 42 adults	[8,16,17,18,19]
Fibrosis, remodeling, and lung repair	siRNA or mRNA targeting TGF-β1, PAI-1/CXCR4, hepatocyte growth factor (HGF), Tbx2, or encoding IL-11 single-chain variable fragment (scFv)	Suppress profibrotic signaling or express repair-associated and antifibrotic proteins	Supported by bleomycin fibrosis, idiopathic pulmonary fibrosis (IPF), emphysema, silicosis, fibroblast, epithelial, macrophage, and lesion-level models	[20,21,23,43,72,73,74]
Inflammatory and immune-cell-directed therapy	siRNA, anti-microRNA (anti-miR), or therapeutic RNA targeting TNF-α, TLR4/NF-κB, interleukin-1 beta (IL-1β), interleukin-8 (IL-8), C-X-C motif chemokine ligand 1 (CXCL-1), GATA3, miR-155, or related inflammatory pathways	Reduce inflammatory signaling or modulate macrophage, T-cell, endothelial, or epithelial pathways	Recurrent use of macrophage, acute lung injury (ALI), asthma, pneumonia, endothelial-targeting, and precision-cut lung slice models	[7,24,25,27,42,63,70,75,76,77]
Infectious disease and mucosal vaccination	siRNA, mRNA, or decoy oligodeoxynucleotide (ODN) targeting SARS-CoV-2 RdRp, SARS-CoV-2 spike, influenza hemagglutinin, or bacterial infection pathways	Block viral replication, express vaccine antigens or antibodies, or modulate host–pathogen interactions	Includes infected-cell systems, Syrian hamster SARS-CoV-2 infection, lethal viral challenge, and nasal or mucosal models	[3,5,28,66]
Lung cancer, metastasis, and tumor microenvironment modulation	siRNA, antisense oligonucleotide (ASO), miRNA mimic, mRNA, or clustered regularly interspaced short palindromic repeats (CRISPR)-related RNA targeting Bcl-2, Mcl-1, KRAS, KRAS G12S, multidrug resistance-associated protein 1 (MRP1), class III beta-tubulin (βIII-tubulin), PLK1, PD-L1, discoidin domain receptor 1 (DDR1), tumor protein p53 (p53), interleukin-12 (IL-12), or microRNA-34a (miR-34a)	Induce tumor-cell killing, suppress resistance, edit oncogenic mutations, or remodel antitumor immunity	Broad target diversity, spanning lung cancer cell systems, orthotopic tumors, metastatic models, and non-small cell lung cancer (NSCLC) models	[1,2,4,30,32,33,39,64,78,79]
Platform, reporter, and compartment-access models	siRNA, mRNA, antisense RNA, or CRISPR-related RNA targeting green fluorescent protein (GFP)/enhanced green fluorescent protein (EGFP), luciferase, NanoLuc luciferase, glyceraldehyde-3-phosphate dehydrogenase (GAPDH), or model splicing systems	Establish pulmonary delivery competence, cell access, expression, or intracellular functionality	Useful for method development but should not be overinterpreted as disease-specific therapeutic validation	[1,9,34,37,47,56,62,80,81,82]

**Table 2:** Formulation-design patterns in inhalable pulmonary RNA nanomedicines.

Carrier Architecture, RNA Loading, and Physicochemical Design Pattern	Pulmonary Formulation Problem Addressed	Design Strategy and Technical Anchors	References
Ionizable LNP composition links RNA encapsulation with inhalation-relevant stability	RNA-loaded LNPs must preserve nanoscale structure and cargo association during formulation and processing.	Ionizable lipid, helper lipid, sterol, PEG-lipid, and buffers are co-optimized. Examples include DLin-MC3-DMA/DSPC/DMG-PEG_2000_/cholesterol at 50:10:3:37 with N/P = 3.25; TG4C/DOPE/cholesterol/DMG-PEG_2000_ at 50:10:38.5:1.5; AX4/DSPC/cholesterol/DMG-PEG_2000_ at 35:16:46.5:2.5; Cas9/sgRNA LNPs below 120 nanometers with near-neutral zeta potential and above 80% encapsulation; and pH 5 citrate, poloxamer 188, and glucose buffer stabilization to preserve RNA encapsulation, particle size, and material recovery.	[4,15,20,43,82]
PEG, helper lipid, and sterol selection create trade-offs rather than universal improvements	Surface stabilization can protect LNPs but may reduce intracellular delivery or alter cargo-dependent behavior.	PEG-lipid level, DOPE/DSPC helper lipid, DGTS substitution, cholesterol, and β-sitosterol tune stability, morphology, and cargo compatibility. Higher PEG-lipid content may reduce size or improve shear resistance and mucus penetration, but excessive shielding may reduce cellular expression; DOPE/DSPC effects depended on RNA modality; and β-sitosterol generated polyhedral morphology.	[3,13,18,45,103]
Electrostatic condensation remains central but requires ratio- and surface-controlled formulation	Cationic carriers compact RNA efficiently but risk aggregation, charge-related instability, and inconsistent comparability.	PEI or oligoethylenimine derivatives, chitosan, dendrimers, cyclodextrins, KL_4_, SP-B fragments, and PEGylated peptides use charge-driven complexation. Anchors include chitosan below 300 nanometers at a 5:1 polymer/siRNA ratio, INU-VS-g-(PMeOx;bAPAE) polyplexes below 30 nanometers at a polymer/siRNA weight ratio of 5:1, cyclodextrin complexes of approximately 200 nanometers, generation 4 amine-terminated TPP-modified PAMAM (G4NH_2_-12TPP) at N/P = 30, PEG(12)KL_4_/mRNA at a 10:1 *w*/*w* ratio, and crosslinked chitosan below 150 nanometers with above 96% encapsulation.	[36,37,49,61,93,96,105,107]
Hybrid and multicomponent systems separate RNA protection from inhalable particle engineering	A single material rarely optimizes RNA loading, structural resilience, and pulmonary processability simultaneously.	PLGA, DPPC, DSPE-PEG_2000_, DOTAP, PEI, lipid shells, polymer cores, and perfluorocarbon emulsions create core–shell, co-entrapped, or emulsion-polyplex carriers. Anchors include approximately 164-nanometer DPPC/DSPE-PEG_2000_ hybrid particles with approximately 99% siRNA encapsulation, PLGA/DPPC particles of approximately 150 nanometers with zeta potential near −25 millivolts, DOTAP contents of 15–25% *w*/*w*, and perfluorocarbon emulsion polyplexes.	[23,35,64,67,81,104]
Biomimetic, surfactant-interacting, and hierarchical interfaces move design emphasis to the lung-facing surface	Pulmonary surfactants, mucus, lining fluid, and cell membranes may alter carrier behavior after administration.	Natural pulmonary-surfactant interaction, clinical pulmonary surfactant containing functional SP-B, Curosurf coating, extracellular vesicles, neutrophil membranes, HA-DA, charge-reversal polymers, and peptide adsorption tune the external interface. RDDsT nanocomplexes illustrate a cationic siRNA-containing core, charge-reversal polymer shell, and electrostatically adsorbed RAGE-binding peptide.	[25,39,42,46,63,108,109]
Dry-powder and solid-state matrices must preserve the nanocarrier, not merely generate respirable particles	Processing can change size, aggregation, morphology, RNA integrity, encapsulation, and redispersibility.	Mannitol, leucine, HSA, trehalose, dextran, lactose, sucrose, pH modifiers, NAC, and hydroalcoholic solvents are used as matrices, additives, or processing media to protect RNA or preformed nanocarriers. Anchors include 50% *w*/*w* leucine for naked siRNA; above 6% *w*/*w* siRNA with HSA and up to 78% intact siRNA retained; thin-film freeze-drying (TFFD) processing of siRNA-loaded solid lipid nanoparticles; leucine at 0–60% *w*/*w* in lipid–polymer nanoparticle (LPN) powders; trehalose/dextran (Tre:Dex) at 10:90 with 40% leucine; and redispersed LNP-siRNA of approximately 150 nanometers with below 5% residual moisture and below 15% RNA loss.	[50,52,54,62,83,97,110,111,112,113]
Quantitative reporting determines whether formulation progress is translationally interpretable	Publications and new formulations do not necessarily establish clinical or manufacturing readiness.	The most informative studies report composition together with size distribution, heterogeneity, aggregation, morphology, RNA integrity, encapsulation, surface properties, material recovery, and stability before and after processing. Review-level sources help frame the field but are not substitutes for primary quantitative formulation data.	[4,11,12,20,35,62,81,82,105]

**Table 3:** Translational roadmap for formulation–processing–device readiness in inhalable RNA nanomedicines.

Stage	Evidence Needed for Maturity	Proposed Go/No-Go Signal	Key Bottleneck Clarified	References
Pre-processing formulation	Particle size, PDI or heterogeneity, morphology, surface charge, RNA encapsulation, RNA integrity, and initial formulation stability	Proceed only if baseline carrier and RNA attributes are defined and reproducible before aerosolization or drying	Biological activity before processing is insufficient without defined critical quality attributes	[4,20,35,52,62,81,105]
Liquid aerosolization	Pre- and post-nebulization particle size, aggregation, encapsulation retention, RNA integrity, volumetric median diameter (VMD) or MMAD, fine particle fraction (FPF) where applicable, and retained bioactivity	Stop or reformulate if actuation causes RNA degradation, aggregation, encapsulation loss, or substantial functional loss	Shear, interfacial stress, and droplet formation can convert an active suspension into a damaged aerosol product	[14,37,55,56,57,58,59,60]
Dry-powder manufacture	RNA recovery, residual moisture, crystallinity or amorphous-state stability, redispersed nanoparticle size, morphology, FPF or MMAD, and retained activity	Stop or adjust the formulation or process if drying causes excessive RNA loss, agglomeration, poor redispersion, or reduced activity	Spray drying and low-temperature drying require formulation-specific excipient and process windows	[50,51,52,53,62,81,97,110,111]
Device compatibility	Device-specific output, emitted or respirable fraction, delivered-dose or output reproducibility where measured, carrier integrity after actuation, and retained function	Do not advance unless compatibility with the intended product device is directly demonstrated	Nebulizers, soft-mist systems, pMDIs, and DPIs are not interchangeable, and performance may vary within one device class	[15,49,56,57,58,60,106,108,116,117]
Storage and reconstitution	Stability after storage, reconstitution behavior where applicable, particle-size recovery, encapsulation retention, RNA integrity, retained bioactivity, and aerosol performance	Stop or redesign if storage preserves appearance but not RNA activity, carrier recovery, redispersibility, or aerosol performance	Fresh-liquid activity does not establish shelf-stable or reconstitutable product readiness	[52,53,54,96,108,111,113]
Translational readiness	Integrated critical quality attribute package, aerosol metrics, retained bioactivity, manufacturing-relevant process rationale, storage strategy, delivered-dose characterization, and intended-device alignment	Advance only when manufacturing, storage, aerosolization, device performance, and pulmonary delivery are characterized as one linked system	Publication volume, reporter expression, or preclinical efficacy alone does not establish regulatory or clinical maturity	[11,12,19,52,57,58,59,62,112,113]

**Table 4:** Post-deposition access patterns linking lung barriers, nanocarrier engineering, and cellular targeting in inhalable RNA nanomedicines.

Post-Deposition Access Domain	Engineering Pattern	Key Access Evidence	Translational Interpretation	References
Mucus and sputum traversal	HA modification, PEG tuning, charge shielding, charge reversal, and mucolysis	HA- and PEG-modified lipoplexes diffused through human mucus, whereas unmodified cationic lipoplexes were trapped; PEG failed to improve transport through complex cystic fibrosis sputum; mucin-stable, NAC-containing, HA-DA-coated, charge-reversal, and redispersed LNP-siRNA systems improved mucus-facing behavior.	Mucus penetration is disease- and surface-dependent; PEG is useful only when its shielding does not compromise cellular uptake, escape, or expression.	[18,26,34,42,54,61,62,63]
Surfactant, air–liquid interface, and lung-interface access	Surfactant coating or interaction, SP-B/KL_4_ motifs, PEG-free lipocomplexes, and helper-lipid–cargo matching	Alveofact coating improved air–liquid interface mucus diffusion and epithelial uptake; natural surfactant reduced dextran-nanogel internalization while preserving silencing, whereas the tested cationic lipid comparator was incompatible; SP-B/KL_4_ systems supported cytosolic delivery or clathrin-mediated uptake; helper-lipid/RNA pairing altered mucus penetration, corona formation, and delivery.	Surfactant and lung-interface interactions must be experimentally tested rather than inferred from buffer-based colloidal stability.	[13,38,40,46,65,80,99]
Lung distribution, retention, and systemic escape	Route optimization, porous particles, lipid–polymer hybrids, local liposomes, and perfluorocarbon systems	Broad multilobar or regional distribution was shown for selected chitosan, hPBAE, and LNP systems; pulmonary retention ranged from beyond 48 h to at least 14 days, whereas naked intratracheal siRNA could rapidly escape into the circulation and accumulate in the kidneys.	Deposition does not equal durable pulmonary exposure; retention, clearance, and systemic escape require separate verification.	[6,21,31,43,64,66,67,68,91,119]
Epithelial access	Chitosan, peptide, cyclodextrin, sulfonium LNP, PACE, and epithelial-selective LNP systems	Increased epithelial uptake, association, or transfection was reported in respiratory epithelium, Calu-3, A549, BEAS-2B, club cells, ciliated cells, and human precision-cut lung slices.	Epithelial delivery is well supported preclinically but still requires stronger validation in human disease barriers and in vivo target-cell compartments.	[5,16,27,37,41,92,93,94,100]
Macrophage and inflammatory-site access	Neutrophil membranes, HA/CD44 targeting, charge reversal, macrophage-directed particles, and surfactant-protein coatings	Inflammatory-site accumulation, macrophage uptake, alveolar-macrophage transfection, CD44-mediated internalization, and macrophage-associated repair signaling were demonstrated across multiple systems.	Macrophage delivery is more translationally informative when uptake is linked to mucus traversal, inflamed-site localization, target modulation, or functional repair.	[8,24,25,40,42,63,74,75,84,85]
Tumor and pathological-niche access	Extracellular vesicle (EV)-inspired transfer, HA dual targeting, perfluorocarbon (PFC) nanocapsules, star polymers, ECL nanoliposomes, and EV delivery	LLX enabled tumor-associated macrophage (TAM)-mediated p53 mRNA relay; EV-encapsulated IL-12 mRNA showed preferential cancer-cell uptake; star polymers accumulated in lungs and silenced tumor targets; PFC nanocapsules reached more than 60% of cancer cells and most type II alveolar and bronchial epithelial cells; ECL liposomes improved fluorescent siRNA delivery 26.2-fold; dual-targeted particles accumulated in tumor cells and inflammatory macrophages.	Compelling preclinical access and targeting evidence remains model-specific and is not equivalent to clinical targeting validation.	[2,10,30,33,39,64]
Non-epithelial pulmonary-cell access	Endothelial-targeted dendrimer–lipids, transferrin targeting, lipopolymer–lipid hybrids, and fibrosis-associated LNPs and polyplexes	Pulmonary access or association was shown for Tie2-positive endothelial cells, activated T cells, fibroblasts, and alveolar epithelial type 2 (AEC2) cells; a related systemically administered hybrid also reached lung endothelial and immune-cell compartments.	Broadens the field beyond epithelial replacement but raises higher demands for cell-specific, route-specific, and functional validation.	[7,9,23,87,118]
Intracellular uptake, release, and escape	pH-sensitive swelling, guanidine penetration, SP-B fusion, lysosomal permeabilization, PEG-length tuning, and degradable release	Energy-dependent or receptor-mediated uptake, cytoplasmic transport, endosomal membrane fusion or pH-sensitive escape, rapid carrier degradation, and lysosomal membrane permeabilization were reported; PEG-chain effects remained formulation-specific.	Cellular association and functional RNA bioavailability are distinct; intracellular release or escape must be directly measured or mechanistically supported.	[20,38,65,71,73,89,90,95,109,117]

**Table 5:** Outcome-centered pharmacodynamic, safety, and translational benchmarks for inhalable RNA nanomedicines.

Benchmark	Evidence Pattern	Key Quantitative or Technical Anchor	Proposed Translational Go/No-Go Implication	References
Post-processing bioactivity	RNA function can survive aerosolization or drying, but preservation is platform- and cargo-specific.	Chitosan/siRNA silencing changed from 68% before to 62% after aerosolization; cyclodextrin complexes retained luciferase knockdown at 39.8 ± 3.6% before versus 35.6 ± 4.55% after nebulization; TFFD preserved siRNA function, whereas mesh nebulization and spray-drying stress produced cargo- or process-dependent losses; spray-dried LNP-siRNA achieved above 90% protein downregulation in vitro and up to 50% GAPDH silencing ex vivo without cytokine elevation.	Advance only if RNA integrity and biological activity are retained after the exact manufacturing and delivery process.	[37,52,55,62,91,110]
Pharmacodynamic potency	Strong knockdown or expression is achievable, but potency varies by carrier, cargo, target, and model.	Above 60% EGFP knockdown; 40–80% in vitro silencing; 88% GATA3 silencing in human lung slices; 80–90% luciferase knockdown after nebulization; 70% Bcl-2 mRNA knockdown; and up to 90% on-target CRISPR editing in vitro.	Prioritize reproducible target engagement in lung-relevant models, not only reporter expression in readily transfected cells.	[4,27,31,36,47,90]
Disease modification	Higher-maturity evidence links RNA-mediated molecular activity to therapeutic outcomes.	TGF-β1 siRNA-LNPs reduced inflammatory infiltration and matrix deposition; GM-CSF mRNA reduced surfactant-protein accumulation more effectively than recombinant GM-CSF; PFC miR-34a doubled median survival versus paclitaxel; late-stage IPF survival, ALI amelioration, silicosis-associated repair, and protection against lethal H1N1 or SARS-CoV-2 challenge were also reported.	Treat disease modification as a higher maturity level than uptake, deposition, reporter expression, or molecular knockdown alone.	[3,5,8,20,23,42,64,71,73]
Pulmonary versus systemic delivery	Local administration can improve lung exposure, reduce off-target distribution, or enhance efficacy relative to systemic dosing.	Inhaled pacDNA required substantially lower dosing than intravenous pacDNA; intratracheal liposomes increased pulmonary retention and anticancer efficacy; inhalation therapy reduced tumor volume by above 90% versus approximately 40% after intravenous doxorubicin; and aerosolized polyester nanoparticles avoided liver accumulation while enabling lung-tumor gene silencing.	Pulmonary delivery is justified when it provides a measurable exposure, distribution, safety, or efficacy advantage over systemic dosing, not simply because the disease is in the lung.	[1,6,69,116]
Carrier-dependent efficacy and safety	Nanocarrier design determines pharmacodynamics, surfactant compatibility, barrier behavior, and toxicity.	Non-PEGylated hybrids outperformed PEGylated systems in complex cystic fibrosis sputum; HA-coated LPD particles reduced cytotoxicity and improved Bcl-2 silencing; dextran nanogels retained silencing after surfactant exposure, whereas the tested Lipofectamine RNAiMAX comparator was incompatible; LNPs achieved up to 93% Fluc knockdown after nebulization versus 30% for PEGylated lipoplexes; and hybrid nanoparticles prolonged silencing while reducing cationic-lipoplex-associated inflammation.	Evaluate carrier engineering through coupled efficacy, barrier performance, and safety endpoints rather than physicochemical optimization alone.	[26,46,60,67,78]
Device and process compatibility	Device choice and manufacturing stress can preserve or destroy RNA-carrier function.	Selected soft-mist, laminar-fluid-ejection, and microfluidic platforms preserved mRNA-LNP or RNA-LNP integrity and function better than conventional nebulization; laminar fluid ejection produced three-fold higher luciferase activity; mesh nebulization preserved siRNA function but reduced mRNA-LNP activity; and aerosolization or drying reduced function in selected A1AT mRNA, mRNA-LNP, and siRNA systems.	Do not advance a carrier without compatibility testing using the intended inhaler, formulation buffer, and manufacturing workflow.	[15,16,53,55,56,57,58,59,110,111]
Safety and immunotoxicity	Favorable tolerability occurs in selected systems, but immune liabilities remain formulation- and dose-specific.	PEG-modified PEI caused severe pulmonary inflammation and bronchoalveolar IgM elevation; chitosan–tripolyphosphate mRNA nanoparticles caused dose-dependent cytokine release; cyclodextrin toxicity appeared at mass ratios of 50–100; other platforms reported low toxicity, no systemic toxicity, or favorable repeated-dose profiles.	Go/no-go decisions require pulmonary immunotoxicity and repeated-dose assessment, not only cell viability, short-term histology, or efficient delivery.	[7,19,20,36,37,41,47,62,68,94]
Clinical translation boundary	Human feasibility has been demonstrated, but meaningful clinical efficacy remains unresolved.	MRT5005 enrolled 42 adults with cystic fibrosis; 14 febrile reactions occurred in 10 treated participants, 2 participants discontinued, 2 hypersensitivity reactions occurred, and no beneficial or consistent FEV_1_ effect was observed.	Clinical translation requires acceptable tolerability together with target engagement or meaningful functional benefit; administration feasibility alone is insufficient.	[19]

**Table 6:** Evidence-to-practice roadmap for responsible translation of inhalable RNA nanomedicines.

Roadmap Stage	Current Evidence Supports	Key Uncertainty	Practice Interpretation	Future Priority
Rationale	Local pulmonary RNA delivery is biologically plausible for diseases in which the target mechanism, RNA modality, and pulmonary cell compartment are clearly aligned.	Which indications offer the best balance of reachable target cells, measurable local benefit, and acceptable repeat-dose risk remains unresolved.	Prioritize indications with a defined molecular defect or pathway and a verifiable lung-localized pharmacodynamic endpoint.	Establish target-product profiles before carrier optimization, including target cell, disease mechanism, dosing logic, and intended clinical endpoint.
Mechanism	Nanocarriers can support RNA protection, aerosol compatibility, mucus or surfactant interaction, cellular uptake, and intracellular RNA activity.	Mechanistic success is often inferred from uptake, reporter expression, or knockdown rather than proven intracellular bioavailability in disease-relevant cells.	Treat deposition, uptake, endosomal escape, target engagement, and therapeutic effect as separate evidence requirements.	Use orthogonal assays that connect extracellular barrier traversal, target-cell access, intracellular release, and RNA-mediated function.
Evidence base	The field shows strong preclinical feasibility across mRNA, siRNA, ASO, miRNA, saRNA, and genome-editing approaches, with several disease-relevant pharmacodynamic signals.	Evidence remains uneven, with many studies centered on reporter systems, short-term models, or single favorable readouts.	Interpret platform studies as enabling evidence, not as proof of therapeutic readiness.	Build staged evidence packages that progress from formulation quality to disease-relevant activity, repeat dosing, and comparative benchmarking.
Populations and contexts	The most compelling contexts are those in which local delivery can plausibly improve exposure, reduce systemic distribution, or access pulmonary epithelial, macrophage, fibroblast, immune, endothelial, or tumor compartments.	Human disease barriers, patient-to-patient mucus or sputum heterogeneity, inflamed tissue access, and clinical target-cell engagement remain insufficiently resolved.	Avoid generalizing from healthy animals, immortalized cell lines, or simplified mucus systems to diseased human lungs.	Expand use of primary human cells, air–liquid interface models, precision-cut lung slices, patient-derived mucus or sputum, and clinically relevant exposure systems.
Outcomes	RNA nanomedicines can produce target knockdown, protein expression, cytokine modulation, fibrosis suppression, antiviral or antibacterial effects, tumor control, and immune activation in selected models.	Durability, dose-response relationships, functional reversibility of disease, long-term remodeling, and clinically meaningful benefit are not yet established for most platforms.	Give greatest weight to studies linking molecular activity to histological, physiological, infectious, fibrotic, oncologic, or functional improvement.	Define outcome hierarchies that distinguish delivery competence, pharmacodynamic activity, disease modification, and clinical benefit.
Practice and implementation	Inhalable RNA products can be formulated as nebulized liquids, soft-mist aerosols, pMDI products, DPI products, and dry powders when processing and device conditions are matched to the cargo–carrier system.	Compatibility with one device, excipient, drying process, or aerosol platform is not transferable across RNA modalities or nanocarrier classes.	Advance candidates only when the intended formulation, manufacturing process, storage condition, and clinical device are tested as one integrated system.	Standardize go/no-go criteria for pre- and post-processing RNA integrity, encapsulation, aggregation, emitted dose, respirable fraction, redispersion, retained bioactivity, and dose reproducibility.
Future gaps	The field has moved beyond asking whether RNA can reach the lung and now has enough evidence to define translational benchmarks.	Pulmonary immunotoxicity, repeat-dose tolerability, scalable manufacturing, clinical-device performance, target-cell engagement in humans, and comparative efficacy remain major bottlenecks.	Classify current progress as scientifically advanced but clinically provisional unless safety, function, access, and manufacturability converge.	Develop consensus translational standards that integrate critical quality attributes, barrier-aware models, immunotoxicity panels, device-specific testing, and clinically anchored efficacy benchmarks.

## Data Availability

No new data were created or analyzed in this study.

## Abbreviations

A1AT	alpha-1 antitrypsin
A549-luc	luciferase-expressing A549
AEC2	alveolar epithelial type 2
ALI	acute lung injury
anti-miR	anti-microRNA
aPAP	autoimmune pulmonary alveolar proteinosis
AQP1	aquaporin 1
AQP5	aquaporin 5
ASO	antisense oligonucleotide
Bac-TMC	baclofen-functionalized trimethyl chitosan
Bcl-2	B-cell lymphoma 2
Cas9	CRISPR-associated protein 9
CD4	cluster of differentiation 4
CD44	cluster of differentiation 44
CFTR	cystic fibrosis transmembrane conductance regulator
COPD	chronic obstructive pulmonary disease
COVID-19	coronavirus disease 2019
CRISPR	clustered regularly interspaced short palindromic repeats
CXCL-1	C-X-C motif chemokine ligand 1
CXCR4	C-X-C chemokine receptor type 4
DDR1	discoidin domain receptor 1
DGTS	diacylglyceryltrimethylhomoserine
DLin-MC3-DMA	dilinoleylmethyl-4-dimethylaminobutyrate
DMG-PEG2000	1,2-dimyristoyl-rac-glycero-3-methoxypolyethylene glycol-2000
DNA	deoxyribonucleic acid
DNAI1	dynein axonemal intermediate chain 1
DOPE	1,2-dioleoyl-sn-glycero-3-phosphoethanolamine
DOTAP	1,2-dioleoyl-3-trimethylammonium-propane
DPPC	1,2-dipalmitoyl-sn-glycero-3-phosphocholine
DPI	dry-powder inhaler
DSPE-PEG2000	1,2-distearoyl-sn-glycero-3-phosphoethanolamine–PEG2000
DSPC	1,2-distearoyl-sn-glycero-3-phosphocholine
ECL	dioleoyl-sn-glycero-3-ethylphosphocholine/cholesterol
EGFP	enhanced green fluorescent protein
ENaC	epithelial sodium channel
EV	extracellular vesicle
FEV1	forced expiratory volume in 1 s
Fluc	firefly luciferase
FPF	fine particle fraction
G12S	glycine 12 to serine
G4NH2-12TPP	generation 4 amine-terminated TPP-modified PAMAM
GABA(B)	gamma-aminobutyric acid type B
GAPDH	glyceraldehyde-3-phosphate dehydrogenase
GATA3	GATA-binding protein 3
GFP	green fluorescent protein
GM-CSF	granulocyte-macrophage colony-stimulating factor
GOCMCS	guanidinylated O-carboxymethyl chitosan
GSD	geometric standard deviation
H1N1	hemagglutinin 1 neuraminidase 1
HA	hyaluronic acid
HA-DA	hyaluronic acid–dopamine
HFA	hydrofluoroalkane
HGF	hepatocyte growth factor
HNP	hybrid lipid nanoparticle
hPBAE	hyperbranched poly(beta-amino ester)
HPLC	high-performance liquid chromatography
HSA	human serum albumin
IgM	immunoglobulin M
IL-1β	interleukin-1 beta
IL-8	interleukin-8
IL-11	interleukin-11
IL-12	interleukin-12
INU-VS-g-(PMeOx;bAPAE)	inulin-vinyl sulfone-graft-poly(2-methyl-2-oxazoline; 1,2-bis[3-aminopropylamino]ethane)
IPF	idiopathic pulmonary fibrosis
IVIS	in vivo imaging system
IVT	in vitro-transcribed
KRAS	Kirsten rat sarcoma viral oncogene homolog
LLX	inhalable liquid-core lipid nanoplatform
LNP	lipid nanoparticle
LPD	liposome–protamine–DNA
LPN	lipid–polymer nanoparticle
LPS	lipopolysaccharide
Mcl-1	myeloid cell leukemia 1
miR-34a	microRNA-34a
miR-145	microRNA-145
miR-155	microRNA-155
miRNA	microRNA
MMAD	mass median aerodynamic diameter
mRNA	messenger RNA
MRP1	multidrug resistance-associated protein 1
N-2-HACC	N-2-hydroxypropyltrimethyl ammonium chloride chitosan
NAC	N-acetylcysteine
N/P	nitrogen-to-phosphate
NF-κB	nuclear factor kappa-light-chain-enhancer of activated B cells
NSCLC	non-small cell lung cancer
ODN	oligodeoxynucleotide
OEI-HD	oligoethylenimine–hexadecyl
O-LhPAE	four-in-one lipidic hyperbranched poly(beta-amino ester)
OMR	antisense 2′-O-methyl RNA
p53	tumor protein p53
pacDNA	polymer-assisted compaction of DNA
PACE	poly(amine-co-ester)
PAH	pulmonary arterial hypertension
PAI-1	plasminogen activator inhibitor 1
PAMAM	poly(amidoamine)
PBS	phosphate-buffered saline
PCD	primary ciliary dyskinesia
PDI	polydispersity index
PD-L1	programmed death-ligand 1
PDMAEMA-POEGMA	poly(2-dimethylaminoethyl methacrylate)-poly(oligoethylene glycol methyl ether methacrylate)
PEG	polyethylene glycol
PEI	polyethyleneimine
PFC	perfluorocarbon
pH	potential of hydrogen
PLGA	poly(lactic-co-glycolic acid)
PLK1	polo-like kinase 1
pMDI	pressurized metered-dose inhaler
ppFEV1	percent predicted forced expiratory volume in 1 s
RAGE	receptor for advanced glycation end products
RDDsT	receptor for advanced glycation end products-directed dual-sensitive transformable
RdRp	RNA-dependent RNA polymerase
RNA	ribonucleic acid
RNAi	RNA interference
ROS	reactive oxygen species
SARS-CoV-2	severe acute respiratory syndrome coronavirus 2
saRNA	self-amplifying RNA
SAXS	small-angle X-ray scattering
scFv	single-chain variable fragment
sgRNA	single-guide RNA
siRNA	small interfering RNA
SOCS1	suppressor of cytokine signaling 1
SP-B	surfactant protein B
SPECT/CT	single-photon emission computed tomography/computed tomography
TAM	tumor-associated macrophage
Tbx2	T-box transcription factor 2
TEM	transmission electron microscopy
Tf-Mel-PEI	transferrin–melittin–polyethyleneimine
TFFD	thin-film freeze-drying
TGF-β1	transforming growth factor beta 1
Th2	T helper 2
Tie2	angiopoietin receptor Tie2
TLR4	toll-like receptor 4
TNF-α	tumor necrosis factor alpha
TPP	triphenylphosphonium
TRAF6	TNF receptor-associated factor 6
Tre:Dex	trehalose/dextran
VE-cadherin	vascular endothelial cadherin
VMD	volumetric median diameter
*w*/*v*	weight/volume
*w*/*w*	weight/weight
XIAP	X-linked inhibitor of apoptosis protein
α5β1	integrin alpha 5 beta 1
βIII-tubulin	class III beta-tubulin
